# Intracellular virion traffic to the endosome driven by cell type specific sialic acid receptors determines parvovirus tropism

**DOI:** 10.3389/fmicb.2022.1063706

**Published:** 2023-01-23

**Authors:** Tania Calvo-López, Esther Grueso, Cristina Sánchez-Martínez, José M. Almendral

**Affiliations:** ^1^Centro de Biología Molecular Severo Ochoa (CSIC-UAM), Madrid, Spain; ^2^Departamento de Biología Molecular, Universidad Autónoma de Madrid, Madrid, Spain

**Keywords:** icosahedral capsid engineering, parvovirus, VEGF, tropism, sialic acid, virus entry and traffic, endosome, capsid structural transition

## Abstract

Parvoviruses are promising anticancer and gene therapy agents, but a deep knowledge of the entry process is crucial to exploit their therapeutic potential. We addressed this issue while attempting to retarget the oncolytic parvovirus minute virus of mice (MVMp) to the tumor vasculature. Residues at three functional domains of the icosahedral capsid were substituted by rational design with peptides competing with the vascular endothelial growth factor. Most substitutions impaired virus maturation, though some yielded infectious chimeric virions, and substitutions in a dimple at the twofold axis that allocates sialic acid (SIA) receptors altered viral tropism. One dimple-modified chimeric virion was efficiently attached as MVMp to α2-linked SIA moieties, but the infection was impaired by the binding to some inhibitory α2-3,-6,-8 SIA pseudoreceptors, which hampers intracellular virus traffic to the endosome in a cell type-dependent manner. Infectious from nonproductive traffic could be mechanistically discriminated by an endosomal drastic capsid structural transition comprising the cleavage of some VP2-Nt sequences and its associated VP1-Nt exposure. Correspondingly, neuraminidase removal of inhibitory SIA moieties enhanced the infection quantitatively, correlating to the restored virus traffic to the endosome and the extent of VP2-Nt cleavage/VP1-Nt exposure. This study illustrates (i) structural constraints to retarget parvoviruses with evolutionary adopted narrow grooves allocating small SIA receptors, (ii) the possibility to enhance parvovirus oncolysis by relaxing the glycan network on the cancer cell surface, and (iii) the major role played by the attachment to cell type-specific SIAs in the intracellular virus traffic to the endosome, which may determine parvovirus tropism and host range.

## Introduction

Engineering capsids is a common strategy to attempt retargeting viral vectors, but it may also provide valuable insights into the complex process of virus entry. Some genetic manipulations pursue enhancing the specificity of oncolytic viruses (Bell and McFadden, 2014; Miest and Cattaneo, 2014), particularly retargeting their anticancer capacity to the tumor vasculature (Rhim and Tosato, 2007; Arulanandam et al., 2015). Neo-angiogenesis, the formation of new blood vessels from existing ones, is involved in tumor progression, and its inhibition is a major current therapeutic choice (Carmeliet and Jain, 2011). Most anti-neo-angiogenesis therapies target the vascular endothelial growth factor (VEGF; Robinson and Stringer, 2001), a homodimer of approximately 45 kDa binding the tyrosine kinase receptors VEGFR-1 (Flt1; 134 kDa) and VEGFR-2 (KDR/Flk-1; 140 kDa) that transduce mitotic and cell migration signals together with the neuropilin co-receptors (NRP-1 and NRP-2) (Ferrara and Adamis, 2016; Apte et al., 2019). Some oncolytic viruses have been directed against VEGF as vectors that express soluble receptors (Thorne et al., 2006; MacLachlan et al., 2011; Yu et al., 2012), repressors of the VEGF promoter (Kang et al., 2008), anti-VEGF antibodies (Currier et al., 2013), or chemokines that decrease VEGF expression (Lavie et al., 2013). However, modifications of structural components of oncolytic viruses specifically directed against the VEGF system have not been previously described.

In this study, we aimed at retargeting the oncolytic *Protoparvovirus* minute virus of mice (MVM) (Cotmore et al., 2014; Pénzes et al., 2020), strain MVMp (Crawford, 1966), against the neo-angiogenic process of tumors. MVM has shown oncolytic properties against diverse human-transformed cells (Mousset and Rommelaere, 1982; Guetta et al., 1986; Rubio et al., 2001; Riolobos et al., 2010; Ventoso et al., 2010; Paglino et al., 2012) and especially also against human glioblastoma stem cells with patient-specific p53 deregulations (Gil-Ranedo et al., 2021). The structure of the MVM capsid was resolved to atomic resolution (Agbandje-McKenna et al., 1998; Kontou et al., 2005). The sialic acid (sia; N-acetylneuraminic acid) glycan serves as an attachment factor and primary receptor for MVM (Linser et al., 1977; Spalholz and Tattersall, 1983; Rubio et al., 2005). At high x-ray resolution, the sia is allocated in a dimple depression at the twofold axis of symmetry of MVMp capsid (López-Bueno et al., 2006) in close proximity to residues determining tropism *in vitro* (Ball-Goodrich and Tattersall, 1992) and pathogenicity in severely combined immunodeficient mice (López-Bueno et al., 2008). In arrays of glycans, the capsid of MVM strains with different tropisms is bound to selective types of sia (Nam et al., 2006; Halder et al., 2014), although it remains unclear how they contribute to the cell type-specific infection.

For infectious cell entry, the incoming MVM virion traffics through the low endosomal pH (Mani et al., 2006), where the capsid undergoes a drastic structural rearrangement that can be traced by the exposure of the flexible N-terminal sequences of the VP1 and VP2 subunits (reviewed in Ros et al., 2017) presumably through the 5x channel (Kontou et al., 2005), which is facilitated by a track of Gly residues (Castellanos et al., 2013). A *de novo* endosomal exposure and cleavage of some N-terminal sequences of VP2 were essential to the onset infection (Sánchez-Martínez et al., 2012), presumably because it allows the externalization of the VP1-specific N-terminal sequence, as it was shown *in vitro* (Farr et al., 2006) and in cells (Mani et al., 2006). The VP1-Nt sequence carries a phospholipase activity to exit the endosome (Zádori et al., 2001; Farr et al., 2005) and nuclear targeting sequences (Lombardo et al., 2002; Vihinen-Ranta et al., 2002) required for the virion to invade the nucleus.

In attempting to retarget MVM to the tumor vasculature, we replaced residues of three capsid functional domains with peptides that block the binding of VEGF to its receptors and co-receptors (Vicari et al., 2011). The entire collection of MVM chimeric genomes was screened for major phenotypes including capsid assembly, infectious virus yield, and tropism. Some infectious chimeras became particularly suitable tools to investigate the role played by capsid–sia contacts on parvovirus tropism determination.

## Materials and methods

### Construction of the MVM-VEbp chimeras

The prototype strain (p) of the *Protoparvovirus* minute virus of mice (Crawford, 1966) was used in this study. With the exception of the deletion in the dimple (see below) and the insertion of the A7R (ATWLPPR) peptide at the N-terminus of VP2 and VP1 (described in Sánchez-Martínez et al., 2012), namely, the *n* series of chimeras, the mutagenesis in the common region of the VP1 and VP2 capsid proteins was carried out by substituting the corresponding wt MVMp sequence for that of 6-7 mer VEbp peptides, as follows: (i) for the manipulations of the dimple at the twofold axis, namely, the *d* series of chimeric viruses, the peptides P (PQPRPLC), N (NIRRQG), and A (ATWLPPR) were inserted by substituting the wt sequence between amino acids I362 and K368 (both included) of VP2 using the following oligonucleotides (5' to 3'): VEG3A (CAGTGGGCGTGGCTGTGGATCTGCTGGAACTTTTGG) and VEG4A (CCACAGCCACGCCCACTGGAAGCCAATGGCAGTGTT) to insert the P peptide; VEG3B (GCCCTGGCGGCGGATGTTATCTGCTGGAACTTTTGG) and VEG4B (AACATCCGCCGCCAGGGCGAAGCCAATGGCAGTGTT) to insert the N peptide; VEG3C (CCTCGGCGGCAGCCACGTGGCATCTGCTGGAACTTTTGG) and VEG4C (GCCACGTGGCTGCCGCCGAGGGAAGCCAATGGCAGTG) to insert the A peptide; and VEG3-DELTADFw (AACACTGCCATTGGCTTCATCTGCTGGAACTTTTGG) and VEG4-DELTADRv (CCAAAAGTTCCAGCAGATGAAGCCAATGGCAGTGTT) to construct the Δ dimple chimera carrying the 3,876–3,898 (I362-K368 residues) nucleotides deletion. In all cases, two overlapping PCRs on the N-terminal and C-terminal sites of the mutation were conducted using the pSVtk-VPs plasmid (Ramírez et al., 1995) as a DNA template. The two final amplicons with modified nucleotides were assembled and further amplified using the flanking oligonucleotides S1 (GGGGAATTCGCTCAAGGGAGCAGACATGG) (introducing an *EcoRI* target site into the sequence) and S2 (GTAACAATTCTAGAAAGTGTGGCTCCG) at the 3,721–3,740 and 4,329–4,355 nt positions, respectively. The *EcoRI*–*XbaI*-digested amplicon was subcloned into the pUC19 plasmid, and then, the *HpaI*–*XbaI* fragment was cloned into the MVMp infectious clone pMM984 (Merchlinsky et al., 1983). (ii) The construction of chimeric viruses at the spike (namely, the *s* series of chimeras) has been previously described (Grueso et al., 2019); (iii) double chimeras at the dimple and the spike (namely, *d, s* series) were constructed using the oligonucleotides VEG5A (CAGTGGGCGTGGCTGAGGGTTTGCATTTGTAAGAATG) and VEG6A (CCTCAGCCACGCCCACTGGACATTCATTTTTCAAATG) to replace spike sequences with peptide P; VEG5B (GCCCTGGCGGCGGATGTTTGCATTTGTAAGAATGCC) and VEG6B (AACATCCGCCGCCAGGGCAATGACATTCATTTTTC) to replace spike sequences with peptide N; and VEG5C (CCTCGGCGGCAGCCACGTGGCTGCATTTGTAAGAATGCC) and VEG6C (GCCACGTGGCTGCCGCCGAGGGACATTCATTTTTCAAATG) to replace spike sequences with peptide A. The S1 and S2 flanking oligonucleotides were used with the same strategy mentioned above, but here, simple dimple mutants cloned in pUC19 (*EcoRI*-*XbaI*) were used as DNA templates for the new PCR reactions; (iv) double chimeras at the dimple and the VP-Nt sequence (namely, *d, n* series) were constructed with a similar strategy using the oligonucleotides NT-PEPC (GCCACGTGGCTGCCGCCGAGGAGTGATGGCACCAGCCAACC) and NT-PEPC2 (CCTCGGCGGCAGCCACGTGGCCATGGTTTGACTGCTTTGCTG) using VPNT-1 (CCGAATTCATCTCGAGGACCTGGCTTTAG) and VPNT-4 (CCGGATCCCCAGTTAACCCCCATTTGTGTT) as flanking primers to insert the A peptide between the methionine 1 and serine 2 of VP2. The final amplicon was cloned into the chimeric pMM984-Pd, pMM984-Nd, and pMM984-Ad using the *XhoI* (2,071 nt) and *HpaI* (3,758 nt) sites.

High-fidelity Platinum^®^Pfx (Invitrogen) and Pwo^®^ (Roche) polymerases were used following the PCR programs of the manufacturer: in brief, 0.5 μg of template DNA, dNTPs (200 μM), modified oligonucleotides (2 μM), and 2.5 U polymerase Pwo; 1 cycle of 2′ at 94°C; 10 cycles of 15” 94°C, 30” at 55°C, and 1′ 30” at 72°C, 1 cycle of 2′ at 94°C, 20 cycles of 15” at 94°C, 33”at 55°C, and 1′ 30” at 72°C (with 5” increment per cycle), 1 cycle of 7′ at 72°C and 5°C. After cleaning the amplicon bands (Wizard SV Gel and PCR Clean-Up System, Promega), they were used as new templates (1 ul) for the second PCR reaction: 1 cycle of 2′ at 94°C, 10 cycles of 15” at 94°C, 30” at 60°C, and 1′ 30” at 72°C, adding flanking nucleotides (S1 and S2) (2 μM) and repeating 1 cycle of 2′ 94°C, 20 cycles of 15” at 94°C, 30” at 60°C, 1′ 5” at 72°C (with 5” increment each cycle), 1 cycle of 7′ at 72°C and 5°C. Restriction enzymes (Roche) were used for 1 h at 37°C and T4 DNA ligase (New England Biolabs) for 14–16 h at 15°C. Genomic plasmids were grown from a single transformed colony of *Escherichia coli* JC8111 (Boissy and Astell, 1985) and Sanger sequenced to verify the genetic constructions and the absence of additional mutations. Plasmids were purified at a large scale with Qiagen Plasmid Maxi Kit (Qiagen) using the manufacturer's protocol.

### Production of MVMp and chimeric viruses

The origin of the A9 ouabr11 mouse cell line, the human-transformed NB324K fibroblast and U373MG glioblastoma cells, and the respective media and culture conditions have been previously described (Gil-Ranedo et al., 2021). MVMp and chimeric viruses were produced by electroporation of NB324K cells with the respective infectious molecular clones following a previously described method (Lombardo et al., 2002). For normal-scale transfections, 3 × 10^6^ cells were electroporated with 10 μg of plasmid, but the growth chimeric viruses with impaired propagation in culture required transfection at a large scale as described (Sánchez-Martínez et al., 2012). Reference viral stocks were harvested at 48 h post-transfection (hpt), and their infectious titers were determined as described below. Then, large viral stocks were prepared by infecting NB324K at a multiplicity of infection (MOI) of 10^−3^ infectious units/cell and culturing for 5–7 days until the cell monolayers showed a moderate cytopathic effect. The total virus was purified by sucrose cushions and cesium chloride equilibrium centrifugation, and DNA-filled virions were pooled, dialyzed against PBS, and kept at −70°C in aliquots following previously described procedures (Lombardo et al., 2002; Gil-Ranedo et al., 2021). Viral stocks were routinely checked for genetic stability in the VP sequence surrounding the inserted peptides to avoid the possible selection of mutants overcoming assembly restrictions (Grueso et al., 2019).

### Virus titration

Infectious viral particles were commonly titrated by a plaque-forming unit (PFU) assay staining with crystal violet and optimized as described (Gil-Ranedo et al., 2018). A more sensitive PFU assay based on immunostaining was used to quantitate small plaques formed by some chimeric virions. For this, inoculated monolayers were incubated 6 days as above; thereafter, the semisolid medium was carefully removed, washed with ice-cold PBS, and fixed with methanol–acetone (1:1) for 8 min at −20°C. Fixed monolayers were washed two times with PBS, blocked for 30 min at room temperature with PBS supplemented with 5% FCS, and then incubated for 90 min at room temperature under shaking with rabbit anti-VPs serum diluted in the same buffer. Finally, the monolayers were washed two times with PBS, incubated with a goat anti-rabbit Ig-peroxidase conjugate secondary antibody, and developed with the 3,3,9-diaminobenzidine (Sigma) substrate solution. Alternatively, to determine viral tropism as shown in **Figure 3**, infectious viral particles were quantitated as immunofluorescence units (IFUs) staining at 24 hpi with the anti-NS1 or the anti-VPs antibody as described (Lombardo et al., 2002; Sánchez-Martínez et al., 2012). The viral cytotoxic capacity was determined by a conventional cell viability assay based on the conversion of 3-(4,5-dimethylthiazol-2-yl)-2,5-diphenyltetrazolium bromide (MTT) to an insoluble formazan product. For this, cell monolayers growing in M96 well plates were washed in PBS and incubated for 2 h at 37°C with 5 mg/ml MTT in PBS, and after extensive washing in PBS, the dye was eluted with dimethyl sulfoxide (DMSO) for 15 min at room temperature in the dark. The absorbance of the samples at 570 nm was measured as optical density (OD) in a CLARIOstar Plus, BMG Labtech apparatus.

Total virions were quantitated by their protein content in 10% SDS-PAGE staining with Coomassie blue with BSA as an internal control (see **Figure 2A**). This method yielded 1.52 × 10^14^ viral particles per mg taking 3,970 kDa as the MW of the T1 MVM virion, based on the size of the VP1 and VP2 protein subunits (Gardiner and Tattersall, 1988), and their assembly stoichiometry (Riolobos et al., 2006). Virions were also quantitated in triplicates by their hemagglutination activity (HA) with mouse erythrocytes at 4°C in U-profile microtest plates (Nunc) as described (Hernando et al., 2000). The HA test shown in **Figure 2C** results in 750 ± 200 HAU/μg for the MVMp and 200 ± 50 HAU/μg for the Nd virions. Infections and binding assays were performed with normalized amounts of purified virions.

### Antibodies

To study the VEGF system, the following antibodies were used: α-VEGF-R1 rabbit polyclonal (ReliaTech, 102-PA20S), α-VEGF-R1 mouse monoclonal (ReliaTech, 101-M30), α-VEGF rabbit polyclonal (BioVision, 5363-100, or BioLegend, 627501), and the α-A7R mouse monoclonal antibody (Sigma, V4758) were originally used to identify the 7-mer ATWLPPR antiangiogenic A7R peptide binding the VEGF receptor neuropilin-1 (Starzec et al., 2006). Recombinant biologically active human VEGF-A (Peprotech 100-20) and recombinant human endogenous soluble VEGFR-1/Flt-1 (sVEGF-R1; ReliaTech S01-010) were used as controls in the binding assays as shown in **Figure 4**.

The collection of anti-MVM antibodies used in this study has been previously described (Sánchez-Martínez et al., 2012; Gil-Ranedo et al., 2015, 2018, and Grueso et al., 2019; Gil-Ranedo et al., 2021) and comprises the following: the α-NS1 polyclonal antibody, α-VPs rabbit polyclonal antiserum raised against denatured VP2, the mouse Mab-B7 that recognizes an epitope at the threefold axes, the α-MVM rabbit antibody recognizing mainly conformational capsid epitopes, the α-2Nt rabbit polyclonal antibody recognizing the N-terminal sequence of VP2, and the α-VP1-Nt rabbit polyclonal antibody recognizing the 143 amino acids N-terminal sequence of VP1 (Cotmore et al., 1999). The mouse anti-EEA1 (BD, Biosciences) was used to stain the early endosomal compartment by IF.

### Immunological methods

For cytometry, fixed and stained cells in suspension were analyzed through a FACSCanto II Flow Cytometer using a minimum of 2 × 10^4^ events for the sample as described (Gil-Ranedo et al., 2021) and analyzed with the FlowJo software (vs). Samples analyzed by immunoprecipitation (IP) were harvested in PBS, clarified, and incubated at 4°C overnight with the indicated antibodies. Then, after 10% Sepharose was added, the mixture was further incubated for 2 h at 4°C under shaking, and finally, the Sepharose was washed three times with PBS, and proteins were recovered with Laemmli buffer for subsequent blot analysis.

Double-label indirect immunofluorescence (IF) was performed with cells seeded onto glass cover slips following described protocols (Gil-Ranedo et al., 2015, 2021). Samples were inspected by epifluorescence imaging using multiphoton confocal laser scanning LSM710 (Zeiss) coupled to an Axio Observer Inverted Microscope (Zeiss). To quantitate the number of endosomes showing accumulation of the incoming viral capsids as clusters (**Figure 6**, Supplementary Figure S3), an area of 80 μm^2^ was placed within the contour of each early endosome (EEA) delineated by specific immunostaining. Then, merged with the images obtained from the anti-MVM capsid antibody staining, the area of minimal through patent capsid accumulation per field of cells was defined as a cluster by visual inspection, and its values of pixels were measured by the RawIntDen. Finally, the number of EEA showing clusters with a RawIntDen value above the defined threshold was recorded with respect to the total number of endosomes per field of cells. Averages and standard errors were obtained from at least 3–4 confocal images per condition. Representative endosomal clustering phenotypes were supported by images collected in the three dimensions (z-tacks) with a 0.8 μm optical sector and a 0.43 μm step size in an LSM710 (Zeiss) microscope (see Supplementary Figure S5).

### Quantitative protein analysis by Western blot

For Western blotting (WB), samples were electrophoresed in minigels (10 × 10 × 0.1 cm) with molecular weight markers (Precision Plus Protein Dual Color Standards, Bio-Rad) and transferred to nitrocellulose (Schleicher and Schuel) as described (Riolobos et al., 2006). The membranes were probed with the α-VP (1/5,000) and the α-NS1 (1/10,000) primary antibodies, developed with the ECL system (Enhanced Chemiluminescence, Amersham). Samples from cells cultured with fetal calf serum showed a VP-like protein species (arrowhead in **Figures 4C**, **5A**, **B**, **7A**, **B**) likely corresponding to bovine parvovirus capsid protein which did not affect MVM assays. For quantitative analysis of protein levels, the membranes were scanned with a densitometer (Amersham Imager 680) in the linear response range, and the relative band intensities were determined with the ImageJ software (1.53e version) following the instructions of the manufacturer (https://imagej.nih.gov/ij/docs/menus/analyze.html#gels).

### Viral genome replication and packaging

**Figure 1:**
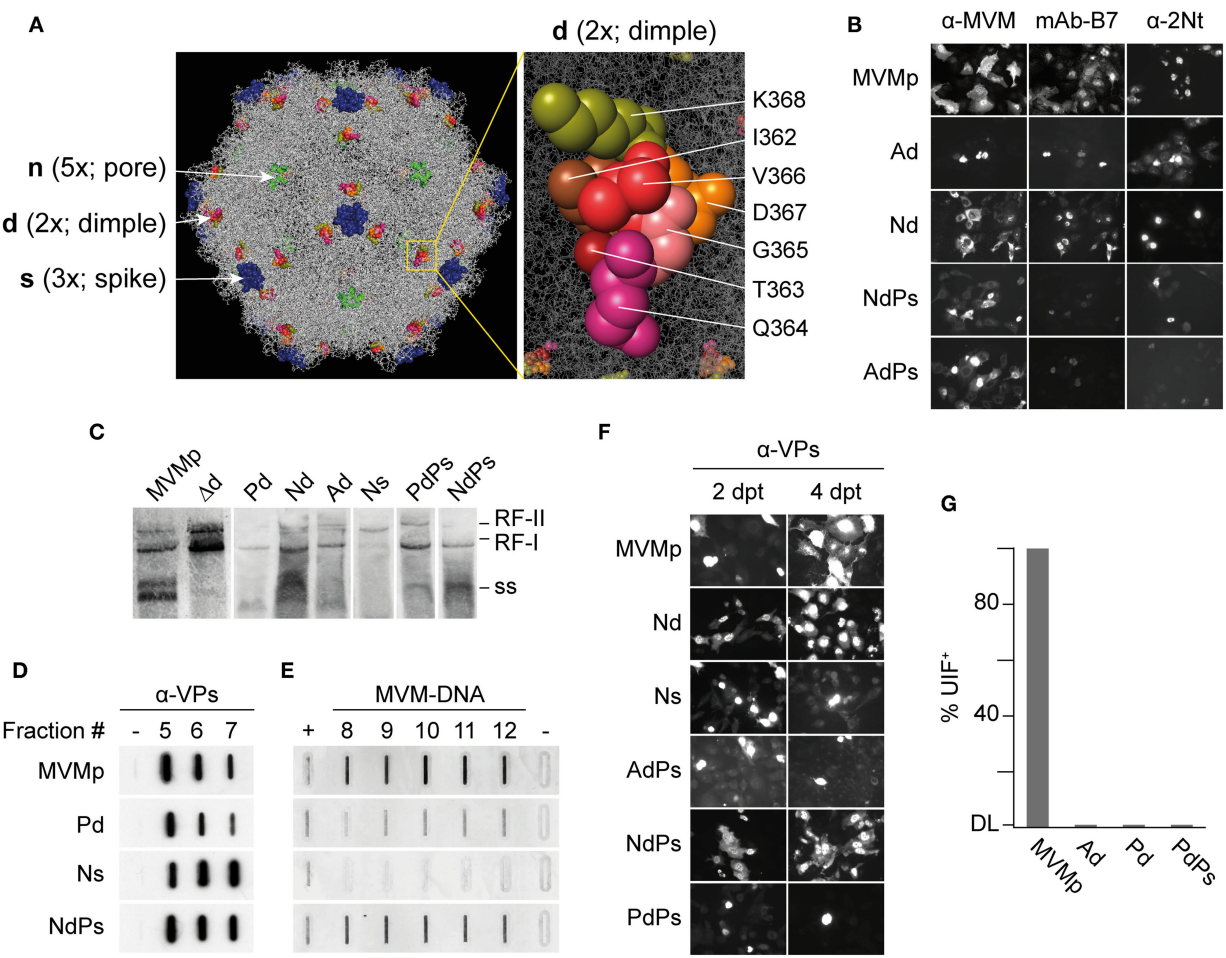
Phenotypes of MVMp chimeras engineered with VEGF-blocking peptides at three capsid functional domains. **(A)** Left: Structure of the MVMp virus capsid (PDB 1Z14) highlighting the domains manipulated with VEbp at the dimple (2x), the spike (3x), and the VP2-N-terminus exposing through the pore (5x). Right: precise set of residues at the dimple (2x) replaced by VEbp. **(B)** IF staining with the antibodies shown above of cultures transfected by the indicated molecular clones (4 dpt). Representative fields are shown. **(C)** Genomic replication and ssDNA encapsidation capacity of chimeric viruses. Southern blot analysis of cultures transfected by the indicated molecular clones (2 dpt). The positions of the single-stranded DNA (ss) virus genome, and that of the monomeric (RF-I) and dimeric (RF-II) replicative forms, are indicated. **(D, E)** Slot-blot analysis of virus assembly performed in cellular samples at 2 dpt fractionated by cesium chloride gradients. **(D)** VPs detected by anti-VPs staining in fractions corresponding to the banding position of the empty capsid. (-), 1 ng BSA as negative control. **(E)** Virus DNA detected by hybridization in fractions corresponding to the banding position of the ss-DNA-filled virion. Controls: (+), 0,05 ng of purified MVMp virion; (-), 1 ng of empty capsid. **(F)** Analysis of chimeric virus progression in culture. Cells were transfected with the indicated genomic clones and stained for VPs expression by IF at 2 and 4 dpt. **(G)** Specific infectivity of some purified virions determined by IF staining of VPs expressing cells at 24 hpi upon inoculation of 12 UHA/10^5^ cells. Results from two experiments with similar outcomes are illustrated. DL, detection limit of the assay.

A DNA slot-blot method was used to study the presence of ssDNA-filled viral particles in cells transfected with genomic clones (Figure 1E). In brief, samples were applied in PBS over nitrocellulose filters using slot-blot filtration manifolds (Hoeffer), and the DNA was denatured by alkaline and hybridized under high stringency conditions with a full-length MVM probe ^32^p-labeled by random priming (Gil-Ranedo et al., 2021). For the analysis of viral DNA replicative intermediates, a Southern blot procedure was used transferring to nylon membranes (Hybond-N +, Amersham Pharmacia) by means of the semidry system (Bio-Rad) at 3.5 mA/cm^2^ for 15 min. The membranes were washed with sodium citrate buffer (0.3 M NaCl and 0.03 M sodium citrate), denatured with 0.5 N NaOH for 5 min, and neutralized with 0.5 M Tris-HCl pH 7.5, and the DNA was fixed with UV light (GS Gene Linker, Bio-Rad) or by heating at 80°C for 2 h in the oven. DNA in the membranes was hybridized to a full-length MVM probe constructed with the DIG High Prime DNA Labeling and Detection Starter Kit II (Roche Applied Science) according to the manufacturer's recommendations, and the color signal was developed using BCIP^®^/NBT (5-bromo-4-chloro-3-indolyl-phosphate/nitro blue tetrazolium chloride) substrate.

### Analysis of the VEGF-R1 use by chimeras

Binding assays were performed with 1.4x10^12^ purified virus particles (6.4 HAU of MVMp and 1.6 HAU of Nd) in 250 μl of PBS supplemented with Ca and Mg ions (PBS^++^) (corresponding to a concentration of 10^−5^ mM of MVM particles) for 30 min at 37°C under shaking with an ~10^4^ molar excess of 0.12 mM (9 μg/mL) or 0.02 mM (1.4 μg/mL) sVEGFR-1 (MW, 72 kDa). These mixtures were inoculated on NB324K and U373MG cells (100 μl/M24 well with approximately 10^5^ cells) for a binding assay carried out for 1 h at 37°C under shaking. VPs and sVEGFR-1 protein levels were determined in the supernatants (unbound) and cell extracts (bound) by WB with the α-VP and the α-VEGFR-1 antibodies, respectively. The Mab-B7 was used as a binding blocking control. The sVEGFR-1 binding activity to its specific VEGF ligand was controlled under similar conditions with 4 μg/mL VEGF and 5 μg/mL sVEGFR-1 and subsequently analyzed by IP with the respective specific antibodies (1/40). To rule out that putative sialic acid moieties in the glycosylated sVEGFR-1 could prevent binding to the Nd chimera, 0,3 μg sVEGFR-1 was treated in duplicate with 2.4 U of NA α2-3, or 15 U of NA α2–3,6.8, in 50 μl PBS^++^ for 30 min at 37°C. Then, 3 × 10^12^ purified MVMp or Nd virions were added in a final volume of 100 μl and incubated for 30 min at 37°C under shaking followed by inoculation onto NB324K and U373MG cell monolayers for adsorption for 1 h at 4°C. Cells were then extensively washed, medium was added, and extracts were collected at 20 hpi in Laemmli buffer to determine NS1 protein levels by WB.

### Sialic acid in virus binding and infection

Virus binding was studied by determining the structural proteins (VPs) bound to cells, and infection by measuring the expression of the nonstructural NS1 protein (see **Figure 5**). The involvement of sialic acid in both processes was determined by treatments with the following neuraminidase (NA): the neuraminidase (acetylneuraminyl hydrolase or sialidase) α2-3 neuraminidase S (NA α2.3 S, New England Biolabs), a highly specific exoglycosidase that catalyzes the hydrolysis of N-acetylneuraminic (or sialic acid) residues linked by α2-3 bonds to other oligosaccharides, and the α2-3,6,8 neuraminidase (2,3,6,8NA; New England Biolabs) that catalyzes the hydrolysis of α2-3, α2-6, and α2-8 linked sialic acid residues. NAs were used with the doses indicated in the figures diluted in 50-μl PBS^++^ per M24 well and incubated for 30–60 min at 37°C while stirring. Then, cell monolayers were inoculated with equivalent amounts of purified MVMp and Nd virions (8 × 10^12^ viral particles per 10^5^ cells) in 50-μl PBS^++^ per M24 well without removing the NAs, and adsorption was left for 45 min at 37°C while stirring. After viral adsorption, the inocula were removed, the corresponding culture medium was added, and the cell monolayers were fixed for IF analysis or harvested in Laemmli buffer at the indicated time points in the figure legends. Control tests performed under similar infection conditions at 5 h post-NA treatments showed that NAs neither affect cell viability nor prevent productive infection (data not shown). The level of VPs and NS1 expression from several independent experiments was quantitated by WB as explained above.

### Statistical analysis

Data were statistically analyzed using the software SPSS (IBM SPSS Statistics 27.0.2) comparing the means of triplicates with an independent samples *t*-test. The equality of variances was analyzed by Levene's test. The significance level is indicated in the figure legends. The results were illustrated in graphics using the software GraphPad Prism 9.

## Results

### Pleiotropic phenotypic effects of VEbp engineered at three functional domains of MVM capsid

The substitution of wt sequences by heterologous peptides of similar size was probed as a better approach than their mere insertion to engineer MVM capsid (Grueso et al., 2019). Thus, in the present study, a peptide substitution approach was followed to attempt retargeting MVM infection to the tumor vasculature. VEGF-blocking peptides (VEbp) of 6–7 mers were inserted by replacing residues of three MVM capsid functional domains: (i) the dimple (2x axis) replacing the residues between I362-K368 (VP2 numbering) involved in direct contacts with sialic acids (López-Bueno et al., 2006); (ii) the loop 4 of the spike at the footprint of the neutralizing B7-Mab (López-Bueno et al., 2003; Kaufmann et al., 2007); and (iii) insertions at the second residue of the VP2 n-terminus, a flexible domain that becomes exposed through the channel at the 5x axis during the entry process (Sánchez-Martínez et al., 2012 and references therein). The inserted VEbp were as follows: (i) the heptapeptide ATWLPPR (A7R or V1) (Binétruy-Tournaire et al., 2000) directly binding to the receptor neuropilin-1 (NRP-1) and thus neutralizing VEGF biological activity (Starzec et al., 2006); (ii) the hexapeptide PQPRPL (P6L) (Giordano et al., 2001, 2005) binding the VEGFR-1 and neuropilin-1 (NRP-1) co-receptors (Pan et al., 2007); and (iii) the hexapeptide NIRRQG (N6G) binding VEGFR-1 with significant affinity (Giordano et al., 2001). For the sake of simplicity in the nomenclature of the chimeras outlined in Table 1, single letters were used to designate both the VEbp as well as the three tackled capsid functional domains.

**Table 1:** Phenotype of the MVM-VEbp chimeras.

	**Mono-domain**							**Bi-domain**									
Chimera	Wt	Δd	Ad	Nd	Pd	As^g^	Ns	Ps^g^	An^h^	Ad Ps	Nd Ps	Pd As	Pd Ns	Pd Ps	An Ad	An Nd	An Pd
^a^Capsid epitopes	+	-	+	+	+	+	+	+	+	+	+	+	+	+	+	+	+
^b^3X epitope	+	-	+	+	+	-	-	-	+	-	-	-	-	-	+	+	+
^c^Capsid yield	+	-	nd	+	+	+/-	+	+	+	nd	+	nd	nd	nd	nd	+	nd
^d^Genome replication	+	+	+	+	+	+	+	+	nd	nd	+	+	nd	+	nd	nd	nd
^e^Virus yield	+	-	+	+	+	+/-	-	+	+	nd	+	-	-	+	+	+	+
^f^Progression in culture	+	-	-	+	-	+/-	-	+	-	-	+	-	-	-	-	-	-

Chimeras are named with a capital letter corresponding to the inserted peptides (A, ATWLPPR; N, NIRRQG; P, PQPRPL) and a lower case letter from the capsid domain (d, dimple; s, spike; n, pore).

a Capsid epitopes IF-stained by the α-MVM polyclonal antibody at 2 dpt.

b 3X epitope IF-stained by the mAb-B7 capsid specific monoclonal antibody at 2 dpt.

c Empty capsids purified by density gradients and developed by slot-blot with the α-VPs antibody.

d RF-I genome replicative intermediates resolved by southern blot at 2 dpt.

e Genome-filled virions stained by IF with the α-VP2-Nt antibody, and/or viral ssDNA genomes detected by hybridization in filters.

f Increasing number of VP^+^ cells along the 2-4 dpt interval.

g From Grueso et al. (2019).

h From Sánchez-Martínez et al. (2012).

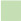
 Full infectious. 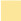 Non-infectious. 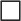 Genome packaging defect.

nd, not determined.

The entire set of genomic molecular chimeras containing single (e.g., Ad) or double (e.g., NdPs) peptide substitutions was transfected in NB324K cells highly permissive to MVMp and analyzed for main virus life cycle stages including capsid assembly, genome replication and encapsidation, virus yield, and the ability to propagate infection to neighboring cells. The representative results of these analyses are shown in Figure 1, and the phenotype of each chimera is outlined in Table 1. All genomic chimeras showed DNA replicative intermediate accumulation (Figure 1C), and capsid proteins (VPs) translocated to the nucleus configuring capsid epitopes (except the Δdimple construct), although the B7-Mab conformational epitope at the 3x axis was not configured in chimeras harboring this epitope replaced by VEbp, as expected (Figure 1B). Some chimeras formed DNA-filled virions (Figure 1D) exposing the VP2 N-terminus (Figure 1B, right) and progressed as infectious entities in culture (Figure 1F), while other chimeric virions were unable to restart infection (Figure 1G). In summary, three major phenotypes could be observed (see Table 1): (i) capsid assembling chimeras, a commonly shared feature with the only exception of the chimera bearing the A peptide in the spike (As); (ii) virion-forming chimeras, eleven of the capsid-forming chimeras allowed viral genome packaging demonstrated by 2Nt-staining (Figure 1B, right), ssDNA resolution in Southern blot (Figure 1C), and slot-blot hybridization of virions banded by CsCl density gradients (Figure 1E); and (iii) infectious chimeras, the DNA-filled virion-forming chimeras that showed the capacity to propagate in culture (Figure 1F) and to initiate infection when inoculated as gradient-purified viral particles (Figure 1G). Therefore, chimeric MVM virions fulfilling infection criteria were the Nd (bearing the N peptide in the dimple), the Ps (bearing the P peptide in the spike), and the NdPs double chimera.

### Replacing dimple residues with VEbp impacts MVM-specific infectivity and tropism

**Figure 2:**
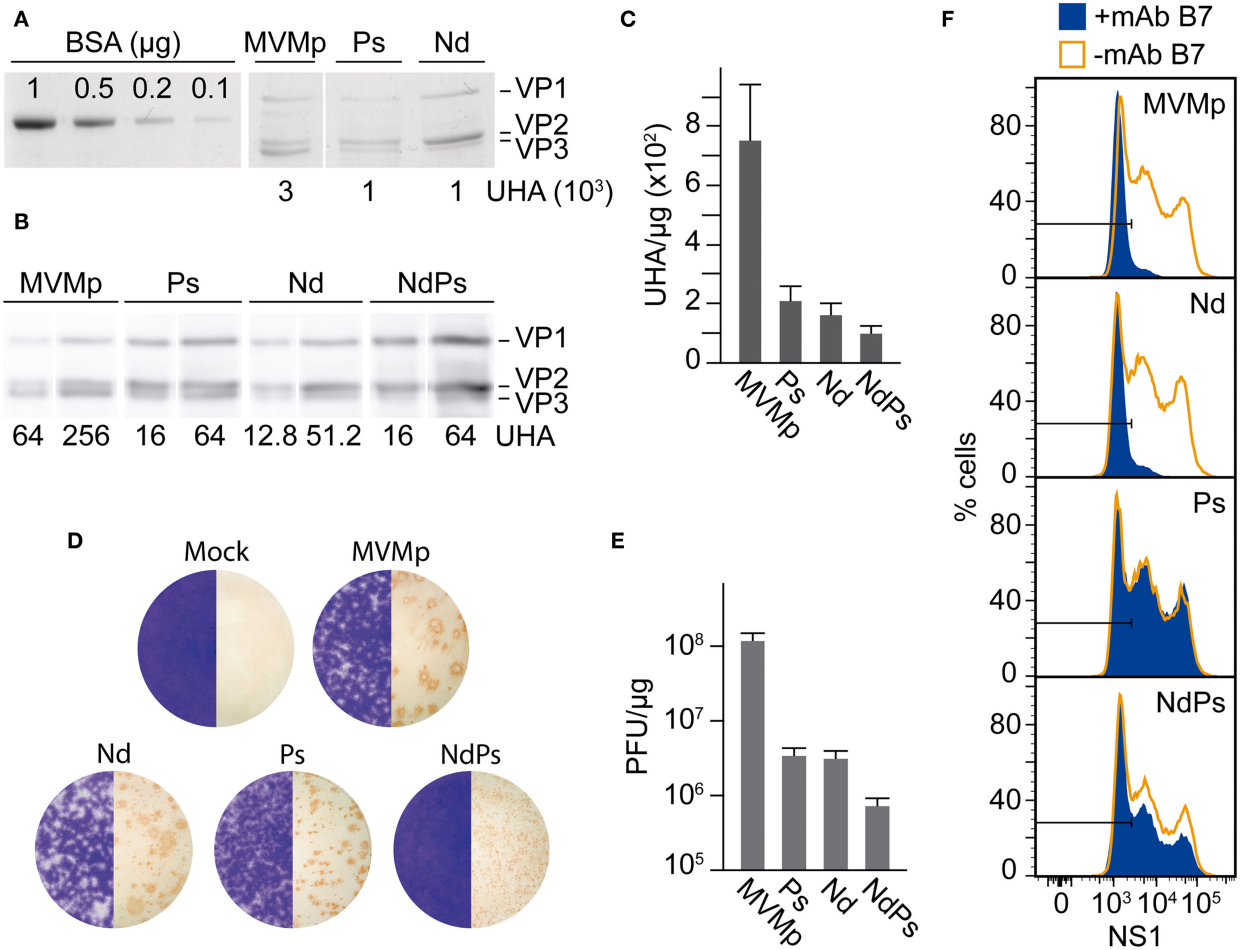
Specific infectivity of MVM-VEbp chimeric virions. **(A)** Quantitative analysis of the hemagglutinating capacity of gradient-purified DNA-filled MVMp and MVM-VEbp chimeric virions determined by **(A)** SDS-PAGE Coomassie staining of VPs from the indicated UHA and **(B)** by Western blot analysis of the virion samples developed with the anti-VPs antibody. BSA (micrograms) was used as mass loading control. The amount of HAUs loaded for each of the viruses is indicated. **(C)** Relative hemagglutination activity obtained from the analysis outlined in **(A, B)**. Values were scored from at least three assays performed with two independent virion preparations. **(D)** Plaque morphology. The figure illustrates the size and lytic character of the plaques formed in NB324K cells by the indicated wt and chimeric viruses. Left half, crystal violet staining using 10^−4^ dilutions of the three chimeras and 10^−6^ of MVMp; right half, anti-VPs antibody staining method (see Materials and Methods) using 10^−4^ dilutions of NdPs, 10^−5^ of Nd and Ps and 10^−7^ of MVMp. **(E)** Specific infectivity outlined as the ratio of PFUs (developed by the anti-VPs antibody staining method in NB324K monolayers) with respect to the mass amount of the inoculated purified virus particles. Values are the means with standard errors from at least three independent determinations. **(F)** Chimeric MVM-VEbp virions evade antibody neutralization. Shown are infected NB324K cultures (MOI 10 using the PFU assay developed by the anti-VPs staining method), incubated with the B7-Mab neutralizing antibody (+mAb B7) or control PBS (-mAb B7) for 20 h, and analyzed by flow cytometry with the anti-NS1 rabbit antibody.

Relevant features of the three infectious MVM-VEbp chimeras were studied with normalized amounts of highly purified virions (Figures 2A, B). The hemagglutination capacity of the chimeras was several folds lower than that of MVMp (Figure 2C), suggesting that the amino acid residues of the dimple and loop four contribute somehow to erythrocyte agglutination. In the inspection of their plaque morphology (Figure 2D), the Nd plaque size was slightly smaller than that of MVMp, Ps formed significantly smaller plaques, and the NdPs plaques were only detected by the anti-VPs antibody staining method. The plaque phenotype reflects restriction in the capacity of the chimeras to assemble and spread to neighboring cells, which paralleled their specific infectivity, 20-fold lower for the single chimeras (Ps and Nd) and two logarithmic units for the double PsNd chimera, with respect to that of the MVMp (Figure 2E). A further relevant feature of these virions was their capacity to evade neutralization (Figure 2F). Consistently, the B7-Mab neutralized the infectivity of Nd but not that of the Ps and PsNd chimeras harboring substituted by VEbp the residues conforming its footprint (Figure 1; Grueso et al., 2019).

**Figure 3:**
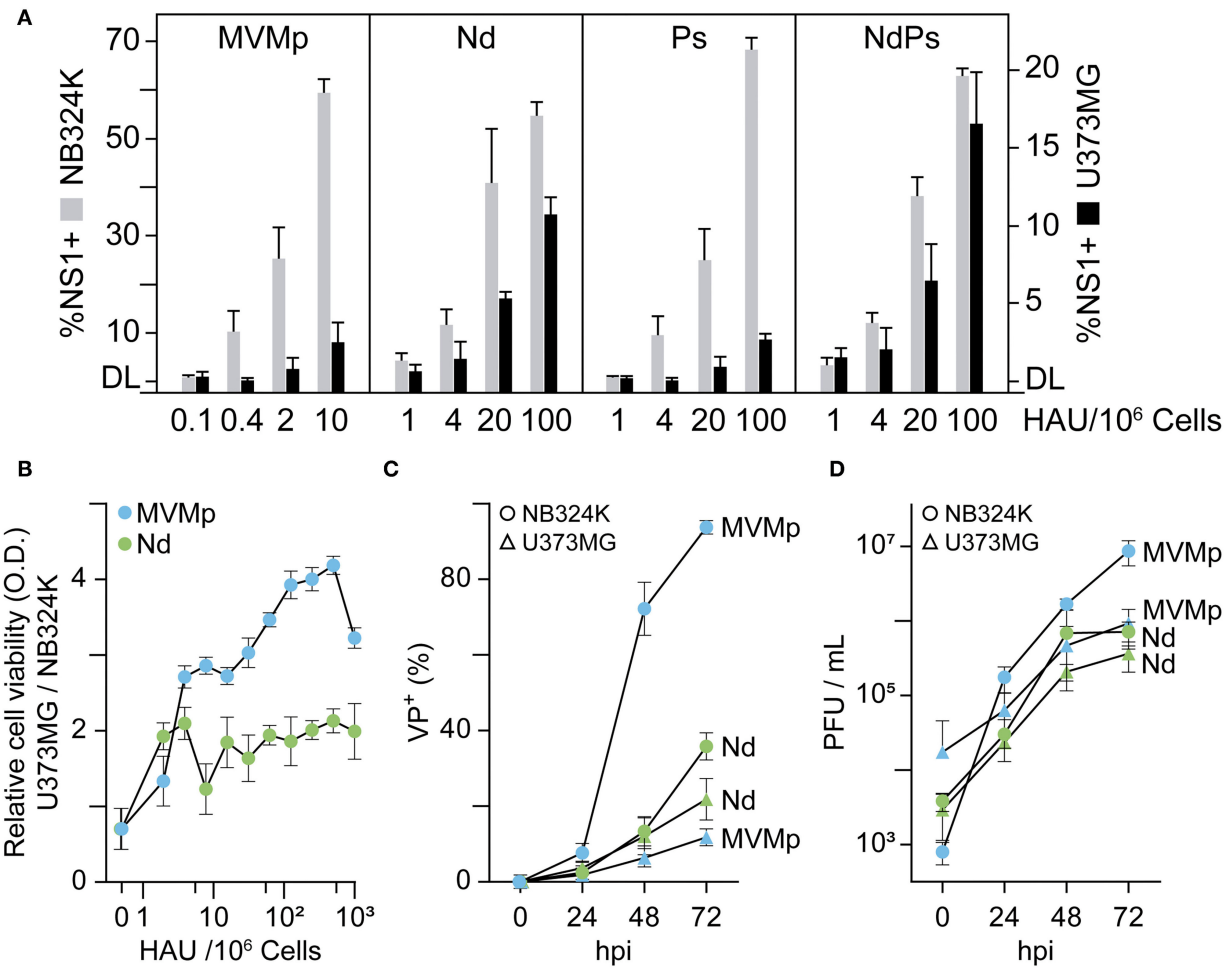
Effect of VEbp insertions on MVMp tropism toward human glioblastoma cells. **(A)** Relative infectivity of MVMp and chimeric Nd, Ps, and NdPs virions in NB324K- and U373MG-transformed human cells. Monolayers of 10^6^ cells were inoculated with the indicated graded amounts of purified virions quantitated as hemagglutination units (HAUs), and the infection was quantitatively scored at 24 hpi by confocal IF staining of NS1 expressing cells. Values correspond to the mean with standard errors of two independent experiments and were obtained by visually counting cells from at least three fields per experiment (n ≥ 10^3^). **(B)** Relative cytotoxic capacity of the MVMp and Nd viruses in U373MG and NB324K cells. Cells seeded onto M96 well plates were inoculated with the indicated serial amounts of purified virions and viable cells determined at 3 dpi by MTT staining. OD, optical density. **(C)** Comparative analysis of the progression of MVMp and Nd infection in U373MG and NB324K cultures inoculated at low multiplicity monitored by IF staining of capsid protein (VP) expressing cells with the anti-VPs antibody. **(D)** Production of total infectious viral particles along the time in cultures infected at low MOI. Values in **(B–D)** are the means with standard errors from three determinations. DL, detection limit of the assay.

Human gliomas express high levels of VEGFR-1 (Plate et al., 1992); therefore, it was of interest to study whether the VEbp substitutions may alter the MVMp natural tropism to glioblastoma (Rubio et al., 2001; Riolobos et al., 2010; Gil-Ranedo et al., 2021). This issue was studied by determining by several methods the infection capacity of the chimeras toward the U373MG human glioblastoma cell line with respect to the reference NB324K human-transformed fibroblasts (Figure 3). While the MVMp-specific infectivity in NB324K determined by NS1 nonstructural protein expression was in the range of ten times higher than that of the chimeras (in consistency with Figure 2E), the NB324K/U373MG-specific infectivity ratio for MVMp and the Ps chimera was close to 30-Fold but only 5-fold for the Nd and the NdPs chimeras (Figure 3A, Supplementary Figure S1). The percentage of NS1-expressing cells consistently correlated with the differential cytotoxicity developed in the cultures, as the U373MG/NB324K ratio of viability was on average 2-fold lower in Nd than in MVMp infection (Figure 3B). The study was supported in cultures infected at low MOI. The progression of MVMp infection (determined by kinetics of VP expressing cells) in NB324K cells was substantially higher than that of Nd, whereas Nd progressed in U373MG cells to levels comparable to that of MVMp (Figure 3C). In consistency, the production of infectious MVMp in NB324K was several folds higher than that in U373MG cells, in contrast to the similar infectious titers reached by Nd in both cell types (Figure 3D). These experiments demonstrated that the chimeric viruses bearing the N peptide substitution in the dimple (Nd and NdPs), but not the one carrying the P substitution in the spike, harbor an increased tropism and cytotoxicity toward the U373MG human glioblastoma cell line with respect to that of MVMp.

### MVM-VEbp chimeric virus infection is not mediated through VEGF-R1

**Figure 4:**
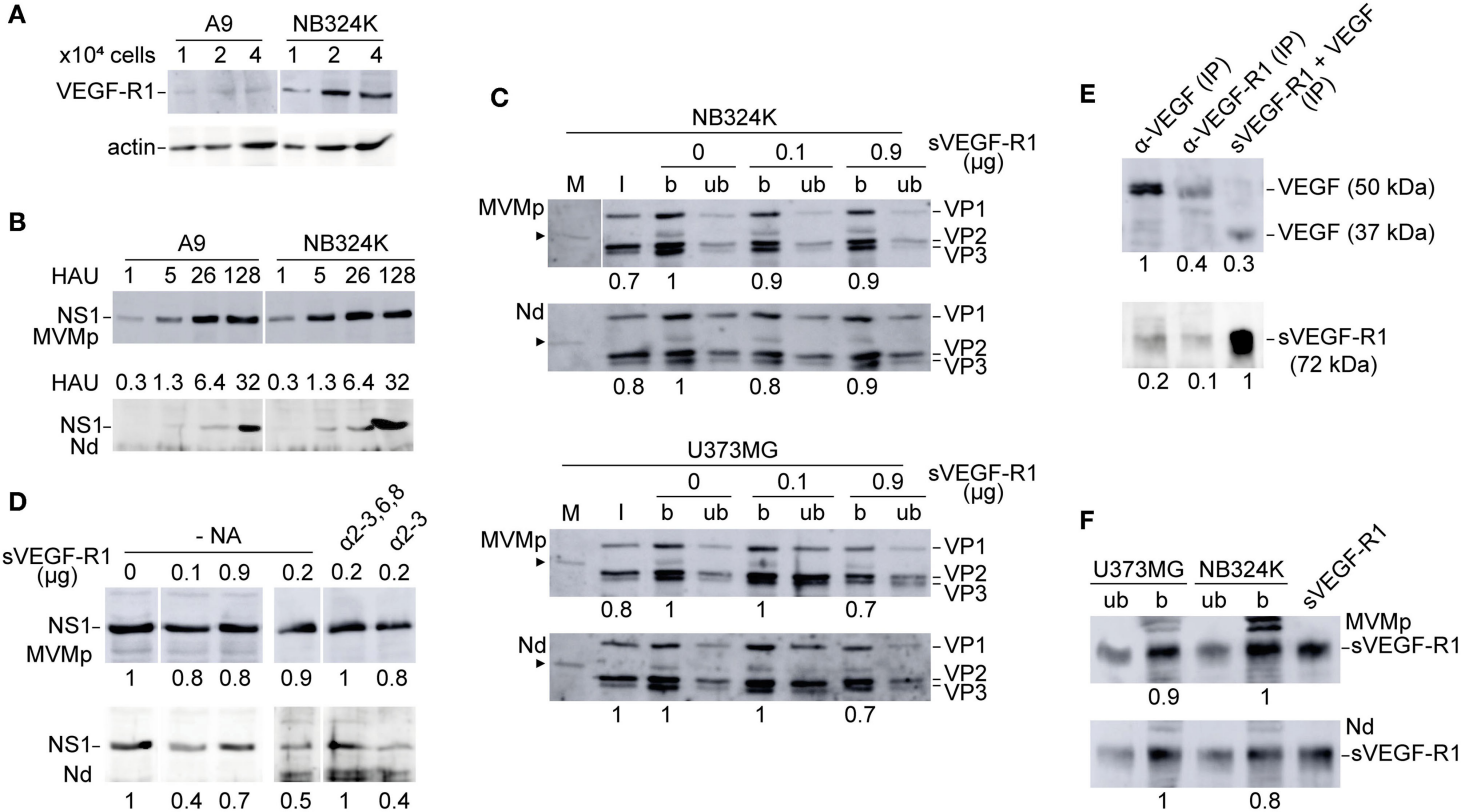
Analysis of the use of VEGF-R1 by the MVMp and MVM-Nd virions. **(A)** VEGF-R1 expression levels in MVMp permissive mouse and human fibroblasts demonstrated by anti-VEGF-R1 polyclonal antibody (Reliatech, 1/200) in WB. **(B)** NB324K/A9 relative infection capacity of the MVMp and MVM-Nd viruses analyzed by NS1 expression at 20 hpi. The number of HAU of purified virions inoculated per 10^5^ cells is outlined. **(C)** Binding analysis of density purified viral particles to NB324K (*upper*) and U373MG (*lower*) cells in the presence of the indicated amounts of sVEGF-R1 and 5x10^11^ viral particles added to 10^5^ cells during virus adsorption. Panels show the viral structural proteins (VP1, VP2, VP3) detected by WB in bound (b) and unbound (ub) samples. Arrowhead marks a VP-related protein present in all cultured cell samples presumably originated from the calf serum. M, Mock; I, input virion. **(D)** NS1 expression in NB324K cells upon virus adsorption in the presence of the indicated amounts of sVEGF-R1 performed as in **(C)**. Right: Similar NS1 expression analysis but following binding at 4°C in the presence of sVEGF-R1 previously incubated at 37°C with 0.05 U/μl of α-2-3 and 0,3 U/μl of α-2-3,6,8 sia cleaving neuraminidases. Shown are WB performed at 20 hpi. **(E)** VEGF/sVEGF-R1 interaction (5 ng/ml each, overnight at 4 °C) analyzed by IP-Sepharose and WB with specific antibodies. **(F)** Control detection of sVEGF-R1 by WB (Reliatech, 1/200) in the b and ub samples shown in **(C)** performed at the higher doses (0.9 μg) of sVEGF-R1. Numbers below the panels are relative arbitrary units (a.u.) of densitometry.

We next sought whether the altered tropism of the MVM-Nd chimera was related to the use of VEGF-R1, as the N peptide inserted at the dimple harbors a high binding affinity to this receptor (Giordano et al., 2001). The expression of VEGF-R1 in cells highly susceptible to MVMp showed high accumulation levels in NB324K cells, although much lower than basal levels in the A9 mouse fibroblasts (Figure 4A). Despite the VEGF-R1 virtually null phenotype, the A9 fibroblasts were susceptible to the Nd infection to levels comparable to that of NB324K (Figure 4B), strongly suggesting that this chimera does not use VEGF-R1 to infect. Furthermore, neither the efficient binding of both MVMp and Nd virions to NB324K and U373MG (Figure 4C) nor the infection of NB324K (Figure 4D) could be significantly decreased with a high excess of soluble VEGF-R1 (sVEGF-R1) as competing decoy added during adsorption. Control experiments ruled out any effect of putative sialic acid moieties of sVEGF-R1 in NB324K infection (Figure 4D, right lanes) and showed the sVEGF-R1 effective binding capacity to VEGF in solution (Figure 4E) as well as to cell surface (Starzec et al., 2006) (Figure 4F). We, therefore, concluded that the Nd virion does not use VEGF-R1 for attachment or infection.

### MVMp and Nd virions attach to different types of α2-linked sialic acids

**Figure 5:**
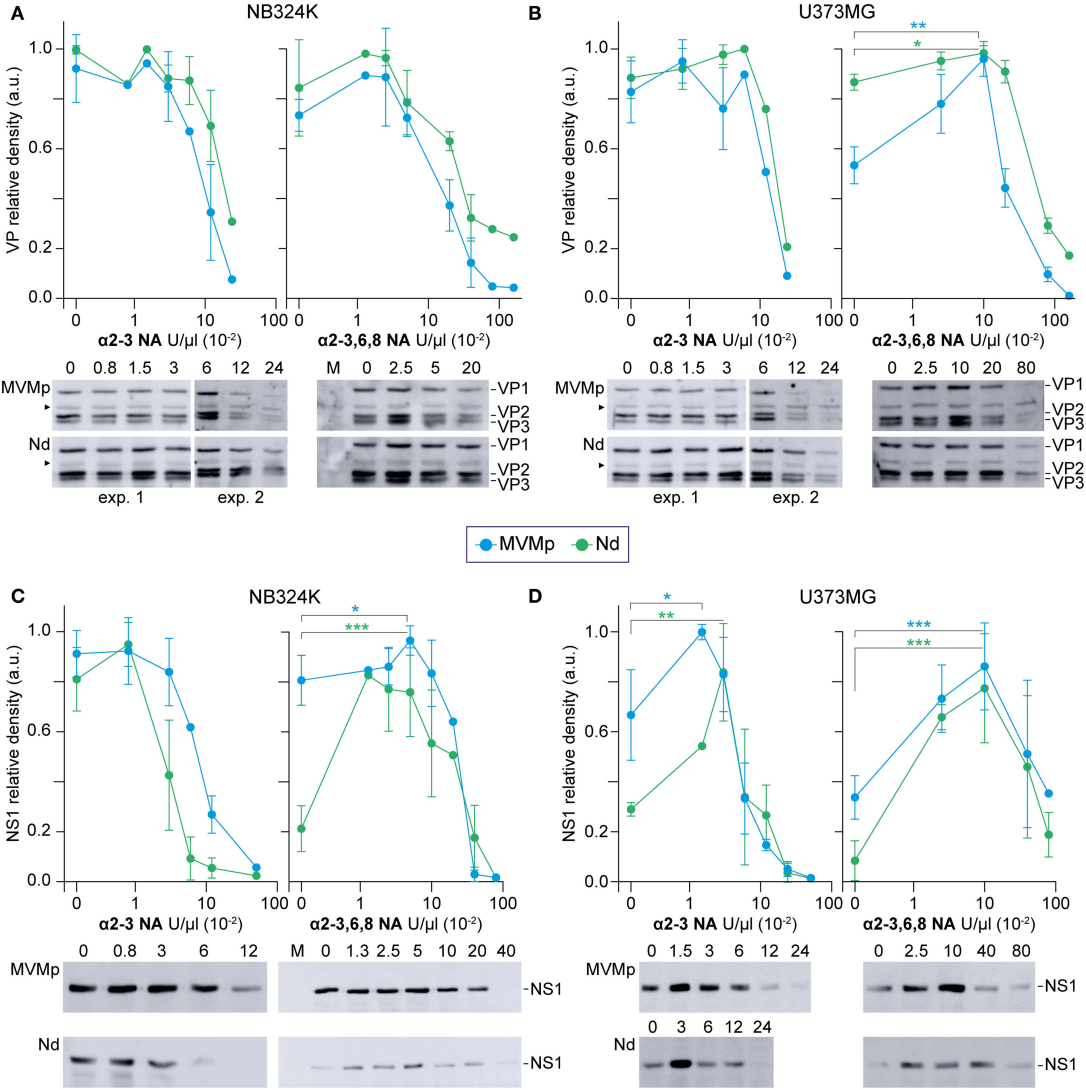
Sialic acids distinctly contribute to MVMp and Nd virions binding and infection of human-transformed cells. The figure illustrates the effect of the α-2-3-NA and the α-2-3,6,8-NA sia cleaving neuraminidases on the binding and infection of MVMp and chimeric Nd virions in human-transformed NB324K fibroblasts and U373MG glioblastoma cells. Cell monolayers were inoculated with equivalent amounts of purified virions (8 x 10^9^ viral particles per 10^5^ cells) in the presence of the indicated concentrations of (left) α-2-3-NA and (right) α-2-3,6,8-NA sia cleaving neuraminidases. Samples were quantitatively analyzed in WB (see Materials and Methods) for: **(A, B)** Binding, developing for the structural proteins (VP1, VP2, VP3) after 1 h adsorption; and **(B, C)** productive infection, developing for the viral NS1 protein expression at 20 hpi. Each experiment was seeded and performed entirely in parallel for the **(A, C)** and **(B, D)** determinations. Each point of the graphs with standard errors represents the average obtained from three to seven independent experiments. Protein density values were normalized in each experiment and means with standard deviations were obtained from the normalized values. a.u. Relative arbitrary units of densitometry. Significance: ^*^p < 0.05, ^**^p < 0.01, ^***^p < 0.001. As above, the arrowhead marks a VP-related protein present in all cultured cell samples.

We next focused on the involvement of sialic acids (sia) in the Nd virion cells interaction, since sia as primary MVM receptor (Linser et al., 1977; Spalholz and Tattersall, 1983; Rubio et al., 2005) allocates in the dimple of the capsid in contact with the I362 and K368 amino acid residues (López-Bueno et al., 2006) that expand the sequence of residues replaced by VEbp (Figure 1A). For this, a quantitative analysis of the attachment to human-transformed cells of purified MVMp and Nd virions was performed under increasing removal of sia moieties from the cell surface with graded amounts of neuraminidases. In NB324K, the α2,3S-NA and the 2-3,6,8-NA gradually decreased the binding since the 5 U/μl doses to a higher extent for the MVMp than for the Nd virion, and even a low proportion of the MVM-Nd virion prevailed bound at the highest doses tested (Figure 5A). In U373MG cells, the α2,3S-NA similarly impaired binding since the 10 U/μl doses with no significative difference between the virions (Figure 5B, left). However, the α2-3,6,8-NA applied at low doses up to 10 U/μl, significantly benefited the attachment of both virions to a higher magnitude for MVMp (Figure 5B, right), suggesting that α2-6, α2-8-linked sia(s) preclude MVMp attachment to U373MG cells. Of note, the general higher resistance of Nd binding to NAs mentioned above was drastically evidenced here, as this virion remained bound at the 20 U/μl doses that sharply decreased MVMp binding, and this tendency prevailed at the highest tested doses of the α2-3,6,8-NA (Figure 5B, right). In conclusion, both virions only use α2-linked sia moieties for quantitative attachment, but the type(s) of α2-6 and α2-8-linked sia usage may differ among them, particularly in the less permissive U373MG cells. Of note, the binding function of the dimple was thus not disrupted by the N peptide, but switched to recognize other types of sia(s). The residual binding capacity of the Nd virus could be accounted by certain marginal types of sia resistant to neuraminidase treatments or by a distinct molecular component of the cell surface to be characterized.

### Binding to sia glycans may inhibit Nd virion infection

A comprehensive comparative analysis of the effect of α2-linked sia removal on MVMp and Nd binding and infection was next performed, the latter measured by the level expression of the major nonstructural NS1 protein. In brief, the kinetics of infection of the MVMp virion in response to α2-linked sia removal with increasing doses of both NAs, respectively, was parallel to those obtained from the binding assays in NB324K (Figure 5A *vs*. Figure 5C), as well as in U373MG cells (Figure 5B *vs*. Figure 5D). Therefore, the MVMp virion binds to the available α2-sia following the multiple NAs treatments to efficiently access the infectious entry pathway in both cell types.

In sharp contrast, binding of the Nd virion to sia moieties did not warrant access to the infectious entry. This remarkable phenomenon was observed under three different patterns of virus–cell interaction. First, the Nd infection in NB324K cells markedly declined with the α2-3NA treatment from 1 to 10 U/μl, despite the high virion binding maintained across this range of doses (Figure 5A *vs*. Figure 5C, left). Second, without NAs treatments, an important relative inhibition of Nd infection with respect to the amount of bound virion occurred in both cell types (Figures 5A, B *vs*. Figures 5C, D). Third, limited removal of sia(s) from the cell surface by NAs sharply increased the Nd infectious entry in most interactions. For example, while the treatment of NB324K cells with high doses of 2,3,6,8NA severely hampered the infection of both viruses to background levels (Figure 5C right, supported by cytometry in Supplementary Figure S2), low doses significantly induced four times the level of Nd infection, but not that of MVMp, a treatment which significantly did not affect attachment (Figure 5A, right). Consistently, the Nd infection in U373MG cells was clearly benefited 3-fold in the α-2,3NA 0-5 U/μl range of doses (Figure 5D, left) and was significantly close to 6-fold in the analysis performed at the 2,3,6,8NA 0-10 U/μl range of doses (Figure 5D, right), despite the efficient virion attachment all across these conditions (Figure 5B, right). Collectively, these experiments demonstrated that α2-linked sia(s) are primary receptors for the infection of the MVMp and Nd virions, but attachment to certain types may severely inhibit access to the infectious entry pathway of the Nd virion in human cells.

### Attachment to sia moieties modulates the traffic to the endosome of the internalized parvovirus virion

The consequences of the differential use of sia by the MVMp and Nd virions across subsequent stages of the entry process were next analyzed. The amount of structural proteins (VP1, VP2, and VP3) bound in adsorption kinetics performed at 4 °C along 100 min showed that the affinity of attachment of the MVMp and Nd virions to the NB324K and the NU373MG cell surface does not significantly differ (Supplementary Figure S3A). The effect of capsid/sia contacts in virus uptake was next analyzed. The MVMp and Nd virions attached at 4 °C could be similarly removed from the surface of both cell types by high doses of the 2,3NA and 2,3,6,8NA neuraminidases (Supplementary Figure S3B, left lanes). However, the NAs were ineffective in releasing the attached virions after 3 h incubation at 37°C that allows internalization (Supplementary Figure S3B, right lanes). This study showed preliminary evidence suggesting that both virions are efficiently uptaken in human-transformed cells.

**Figure 6:**
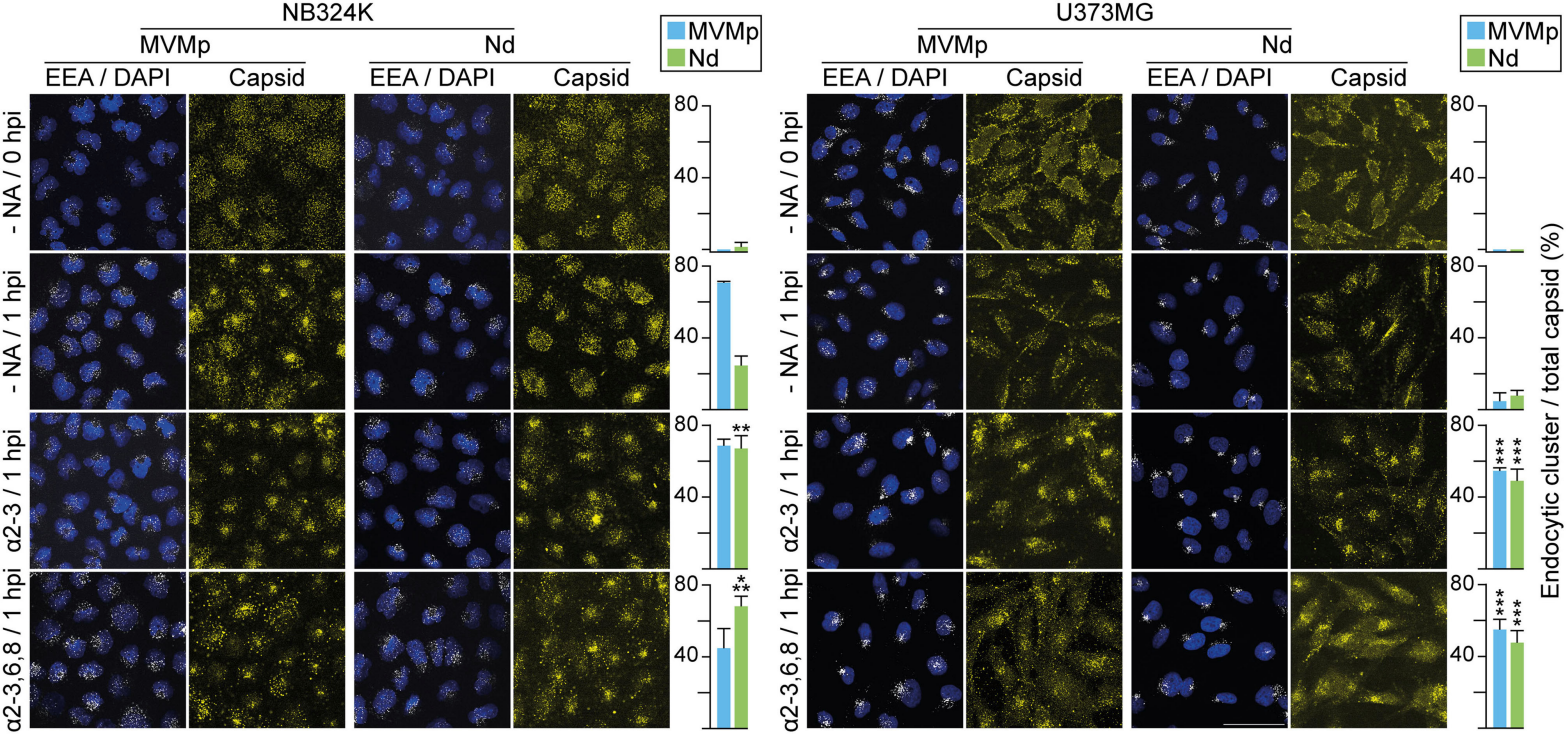
Capsid contacts with sia(s) modulate intracellular parvovirus traffic to the endosome. Effect of capsid–sia contacts in viral traffic to the endosome. The figure illustrates confocal IF staining of MVM capsid (α-MVM capsid polyclonal antibody) and the early endosomal (mouse anti-EEA1 antibody) of cells inoculated with equivalent amounts of purified virions (8 × 10^9^ viral particles per 10^5^ cells) adsorpted at 4°C (0 hpi) and further incubated 1 h at 37°C (1 hpi). Treatments were performed with 2.5 × 10^−2^ U/μl of α-2-3-NA and 10 × 10^−2^ U/μl of α-2-3,6,8-NA sia cleaving neuraminidases. The number of endosomes showing accumulation as clusters of MVM capsid was quantitated as explained in Materials and Methods. Values correspond to the mean with standard errors obtained from three fields (*N* = 10^2^ cells). Statistics was obtained comparing the untreated to the NA-treated cell monolayers. Significance: ^**^p < 0.01; ^***^p < 0.001. Scale bar, 50 μm.

To monitor the effect of sia attachment on cytosolic traffic of the internalized virions, their endosomal accumulation was quantitatively determined by confocal IF staining following NA treatments. Virions attached at 4°C failed to reach the endosome (Figure 6, upper panels), but inspection upon 1 h at 37°C showed that the MVMp virions had efficiently reached most NB324K endosomes, whereas the endocytic Nd accumulation was moderated (Figure 6, left). In the U373MG cells though, the intracellular traffic of both virions was severely restricted as judged by the scattered cytosolic capsid phenotype dominating over the endosomal accumulation (Figure 6, right), in fair correspondence with their specific infectivity in both cell types (Figure 3). However, when U373MG cells were pretreated with the α-2,3 or the α-2,3,6,8 NA, the intracellular traffic of the uptaken virions was drastically shifted, as both efficiently formed endocytic clusters (Figure 6, bottom right). The NA treatments also significantly enhanced the Nd endocytic clustering in NB324K cells (Figure 6, bottom left). Similar results were obtained in the analysis of virus traffic performed at 4 hpi (Supplementary Figure S4), further supported by high-resolution confocal z-stack analysis (Supplementary Figure S5). Importantly, the NAs patent effect on virion endocytic accumulation correlated with their capacity to benefit the infection in both cell types (Figure 5). These experiments provided compelling evidence that the inhibition of the infection originated by the capsid attachment to certain sia on the cell surface is not exerted at the viral uptake step, but rather in the subsequent traffic of the internalized virion to the endosome.

### Capsid–sia contacts determine the extent of endocytic VP2 cleavage and VP1 exposure

**Figure 7:**
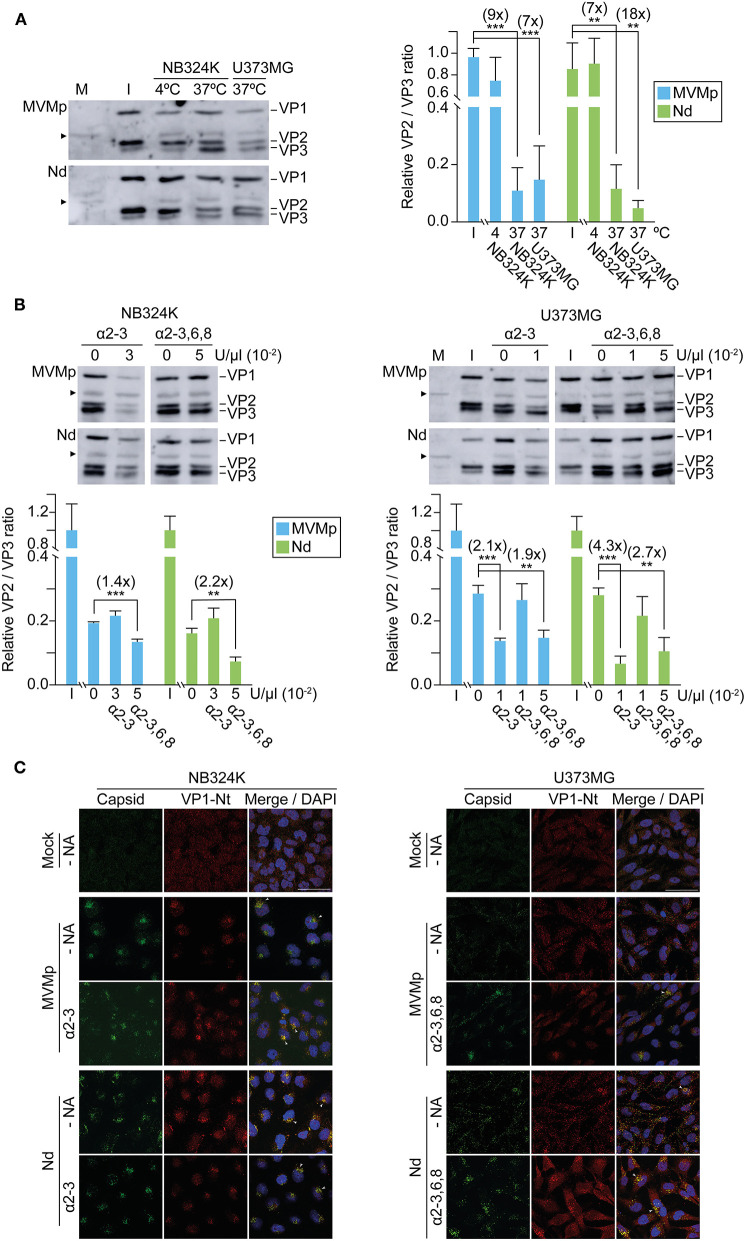
Capsid binding to surface sia moieties influences the extent of endocytic VP2 cleavage and VP1 externalization. **(A)** Different VP2 to VP3 processing of MVMp and the Nd chimera in NB324K and U373 MG cells. Left: Representative Western blot analysis of the VP2 to VP3 processing at 4 hpi. Viral structural proteins (VP1, VP2, and VP3) are indicated. Right: Quantitative values of the VP2/VP3 processing in each cell type. **(B)** Influence of cell type-dependent sia contacts in VP2/VP3 processing at 4 hpi. *Upper:* Representative Western blot analysis of the VP2 to VP3 processing in the presence of the indicated U/μl of NA. *Below*: VP2/VP3 ratio obtained under the indicated NA treatments. Numbers in parenthesis are fold increase. Independent viral stocks with distinct VP2/VP3 ratio were used in the experiments outlined in **(A, B)**. Values were obtained by densitometry and normalized with respect to the VP2/VP3 ratio of the respective input virions (I) in **(A)**, or with respect to the untreated samples in **(B)**. Shown are the means with standard error from three to five independent experiments. Significance: **p < 0.01; ***p < 0.001. **(C)** Sia contacts influence the endosomal exposure of VP1-Nter. Cells were inoculated with purified virions and subcellular localization of viral capsids (B7-mAb antibody), and VP1-Nt exposure at 4 hpi was determined by confocal IF. Viral inoculations and NA treatments were performed as in Figure 6. Arrowheads mark VP1-Nt staining in virions clustering in endosomes. Scale bar, 50 μm.

As a subsequent step of the entry process ensuing virus traffic to the endosome, we next quantitatively analyzed the role of capsid–sia recognition in the VP2 to VP3 cleavage required to initiate the infection (Sánchez-Martínez et al., 2012; reviewed in Ros et al., 2017). The VP2/VP3 processing in the MVMp and Nd virions bound to NB324K cells required incubation at 37°C (Figure 7A), marking an energy-dependent step of the internalization process. With respect to the input virions, while VP2 cleavage was similarly high in NB324K, the extent of the VP2/3 cleavage in U373MG cells was significantly higher for Nd than for the MVMp virion (Figure 7A, right), fairly correlating with the relative tropism of both viruses in these cell types (Figure 3). This experiment showed the relevance of the endosomal VP2/3 capsid cleavage in MVM cell type-dependent infection.

A possible effect of capsid–sia contacts on the VP2/3 cleavage was explored with NAs. In NB324K cells, whereas the NA α-2,3 S did not increase VP2/3 cleavage, the NA α-2,3,6,8 did induce a significant VP2/3 cleavage which was higher in the Nd interaction (Figure 7B, left), correlating with the levels of NS1 expression in these experimental conditions (Figure 5C). In U373MG cells, both the α-2,3 S and the α-2,3,6,8 NAs did significantly increase the VP2/3 cleavage to higher values for the Nd than for the MVMp virion (Figure 7B, right), in tight correlation with the respective benefits observed in the NS1 gene expression of both viruses by NAs in GBM cells (Figure 5D). These results consistently demonstrated that the modulation of MVM tropism by capsid–sia contacts quantitatively correlates with the extent of the VP2 to VP3 cleavage required for infection onset.

The endosomal VP2/3 cleavage can be traced *in vivo* by the associated externalization of the VP1-Nt sequence out of the coat (Farr et al., 2006; Mani et al., 2006). Figure 7C shows viral capsids dispersed in the cytosol, in contrast to the VP1-Nt staining exclusively localized in the capsids accumulated as endosomal clusters; thereby, VP1-Nt exposure was evident in the productive interaction of both virions with NB324K but severely restricted in U373MG. In consistency with the above, the NAs treatments increased VP1-Nt staining, particularly the α-2,3,6,8 NA in the Nd infection of U373MG cells (Figure 7C, below right). Collectively, these experiments demonstrated that capsid contacts with cell surface sia moieties may hamper the traffic of intracellular virions, which precludes a capsid structural rearrangement in endosomal clusters—comprising a *de novo* VP2/3 cleavage and associated VP1-Nt exposure—required to onset infection, but this sia-mediated inhibition may be relieved by mild neuraminidase treatments.

## Discussion

A better understanding of the effect of receptor usage on infectious entry may greatly benefit retargeting vectors and oncolytic parvoviruses to the desired physiological and cancer cell types. This issue was addressed here by attempting to retarget the oncolytic parvovirus MVM (Gil-Ranedo et al., 2021 and references therein), to the tumor vasculature by rationally designing three capsid functional domains with heterologous VEbp. The chimeric MVM-VEbp capsids and DNA-filled virions obtained were analyzed for infectivity, tropism, and receptor usage in two human-transformed cell lines. The approach, aiming at endowing MVM with antiangiogenic properties, uncovers the key effect of sia–capsid contacts on parvovirus cell entry.

### VEbp replacement of capsid residues in MVM assembly and infection

The exquisite structure–function properties of the T1 icosahedral parvovirus capsid impose severe restrictions on exogenous peptide insertions (Carreira et al., 2004), although some insertions were successful (reviewed in Grueso et al., 2019). To circumvent this structural restriction, we replaced short sequences at three MVM functional domains with VEGF-R1 binding peptides, an approach that respected the tight requirements of folding and stoichiometry of VP subunits for the nuclear translocation competence of the assembly intermediates (Lombardo et al., 2000; Riolobos et al., 2006). This rational design approach further illustrated a remarkable tolerance of nuclear capsid assembly to substitutions of residues in the dimple (2x axis) by the three exogenous peptides (P, A, N), whereas the tolerance in the spike (3x axis) was limited to peptide P. The interdigitation of the VP subunits in the spike (Agbandje-McKenna et al., 1998; Kontou et al., 2005) may impair functional insertions, although the virus may evolve by overcoming assembly defects at this site (Grueso et al., 2019).

Despite capsid assembly tolerance, the VEbp peptides may impair infectivity at various stages of the virus life cycle (see Table 1). Some VEbp substitutions (e.g., Ns and PdAs) hampered virus maturation, a defect referring to the process of ssDNA packaging into virions deserving further research. Other VEbp insertions at the Nt domains of the VP1 and VP2 subunits yielded virions devoid of infectivity (e.g., AnNd), a defect that can be accounted for by the requirement of exposure of both Nt sequences through the 5x pore to initiate infection (Sánchez-Martínez et al., 2012). To sum up, the genetic engineering of MVM with VEbp provided valuable insights into capsid structure–function properties including assembly, maturation, and entry. Furthermore, the viable NdPs harboring simultaneously manipulated tropism determinant and immunogenic domains illustrates the possibility to develop infectious chimeric parvoviruses that allow retargeting attempts while evading antibody neutralization in systemic therapies.

### Structural restrains to retarget parvovirus tropism

The VEbp substitution approach eventually yielded the infectious Nd, Ps, and NdPs chimeric virions (Figure 2) which analyze tropism. The identical NB324K/U373MG infectivity ratio of the Nd and NdPs chimeras and that of the Ps and MVMp, respectively, suggest that the spike of the capsid does not determine tropism. In contrast, the Nd and NdPs virions carrying substituted the 362-368 VP2 residues by VEbp showed altered tropism characterized by their relative specific infectivity and toxicity in these cell lines (Figure 3). This is in consistency with the localization of these residues within the dimple, a domain at the 2x axis of the capsid acting as the major determinant of MVM tropism in mice (López-Bueno et al., 2006, 2008). However, none of these chimeric viruses used VEGFR-1 neither for binding nor to infect MVM permissive cells (Figure 4), despite the high inherent binding affinity to VEGFR-1 of the P and N peptides (Giordano et al., 2001), indicating that the depressed configuration of the dimple distorts the functional configuration of the exogenous peptides.

Our study on the Nd tropism for glioblastoma cells has limitations worth mentioning. First, whereas the NB324K/U373MG infection ratio points at Nd as a more specific anti-glioblastoma oncolytic virus than MVMp, the latter harbors a higher absolute infection capacity than Nd to infect the U373MG glioblastoma cell line (Figure 3). Thus, tropism as well as absolute lytic capacity would be key features when considering the development of the Nd virus in cancer therapy in place of MVMp. Second, our study was limited to one GBM cell line, the U373MG, which does not recapitulate the genetic heterogeneity of human primary GBM stem cells and their complex responses to the MVM infection that we have recently described (Gil-Ranedo et al., 2021). Therefore, further research is required with primary GBM cultures and cell lines at the preclinical level to further analyze in depth the anticancer features of the Nd virus.

However, the restriction to retarget MVM with exogenous VEbp is consistent with the encountered difficulties to retarget the parvovirus adeno-associated virus (AAV) with exogenous peptides (Allaume et al., 2012; Castle et al., 2016). The technology of modifying AAV capsid has generated a wide range of vectors with altered tissue tropism or antigenic profile (Li and Samulski, 2020; Liu et al., 2020), which are currently involved in multiple clinical trials. Rational design (Münch et al., 2013; Frederick et al., 2020), directed evolution (Deverman et al., 2016; Körbelin et al., 2016; Davidsson et al., 2019; Tabebordbar et al., 2021), or recombination of serotypes (Grimm et al., 2008; Asuri et al., 2012) are major strategies used to alter AAV tropism. However, experimental evidence on direct AAV capsid–receptor interactions is rare, and therefore, it is unclear whether the actual receptor used *in vivo* matches the originally intended target. With the exceptions of those AAV vectors carrying peptides containing the short RGD motif that recognizes integrins (Girod et al., 1999; Shi and Bartlett, 2003; Tabebordbar et al., 2021) or are tolerated in certain domains (Shi et al., 2001), heterologous peptides inserted in the AAV capsid fail retargeting transduction through the desired receptors (Grifman et al., 2001; Asokan et al., 2010; Büning and Srivastava, 2019). Thus, retargeting MVM to a particular protein receptor remains a difficult aim, and the narrow configuration of the dimple evolutionarily adapted to recognize small glycans as attachment factors and receptors would most likely disrupt those contacts involved in a particular ligand–receptor interaction.

### Attachment to sia pseudoreceptors hampers parvovirus infectious entry

Regardless of their different tropism, the chimeric Nd as the MVMp virion uses only sialic acid receptors, since binding and infection of both virions were fully sensitive to high doses of NAs in the NB324K and U373MG cell types (Figure 5). However, the distinct responses in these extensive quantitative assays performed with a wide range of doses of α-2,3 S and α-2,3,6,8 NAs indicate that these virions use different types of sia glycans for binding and infection, which may account for the relatively higher tropism of Nd for the U373MG cells (Figure 3) since GBM cell surfaces express high levels of aberrant sialylated glycolipids (Yeh et al., 2016; Fabris et al., 2017). Thereby, a major insight derived from these quantitative analyses was the demonstration of the involvement of sia pseudoreceptors for attachment but not for infectious entry. This was clearly evidenced in the Nd/U373MG interaction, where the level of infection could be significantly induced with moderate doses of NAs, a treatment which paradoxically did not increase attachment (Figure 5B *vs*. Figure 5D), strongly suggesting that α2,3, but mainly α2,6- and α2,8-linked sia(s), may act as pseudoreceptors for the Nd virion. In NB324K cells, only α2,6- and α2,8-linked sia(s) pseudoreceptors impair Nd infection (Figure 5A *vs*. Figure 5C). Importantly, the relief of nonproductive binding by lowering the complexity of the sia pseudoreceptor network on cancer cell surfaces may enhance parvovirus oncolytic properties. Yet, extensive delivery trials into solid tumors of free or matrix-embedded virus neuraminidase mixtures will be required to bring this treatment to the clinical context.

A basic sia receptor was resolved to high resolution allocated in the dimple of the MVM capsid (López-Bueno et al., 2006), and correspondingly, MVM strains with changes in dimple residues bound to different types of sia in glycan microarrays (Nam et al., 2006), although the main recognition of the 3'SIA-LN glycan motif did not explain the viral tropism for susceptible cells (Halder et al., 2014). The present study suggests that the characterization of the sia(s) binding capsids *in vitro* may not inform on their role in the infectious entry, as an efficient viral attachment to some types of sia may instead inhibit infection (Figure 5). Many parvoviruses used different types of sia as attachment factors and receptors (Walters et al., 2001; Di Pasquale et al., 2003; Schmidt et al., 2006; Weller et al., 2010; Allaume et al., 2012; Pillay et al., 2016; Pillay and Carette, 2017; Kulkarni et al., 2021), and a failed correspondence between capsid binding and virus infection has been reported in several systems (Schmidt and Chiorini, 2006; Wasik et al., 2016; Callaway et al., 2017), suggesting that sia pseudoreceptors may play relevant common roles in the biology of parvovirus infections.

### A major viral tropism determination: Sia guidance of the incoming virion to the endosome

It could be hypothesized that functional receptors leading to infection are masked by NA-sensitive pseudoreceptors that establish a profusion of contacts with the capsid on the cell surface restricting virion uptake. This possibility was ruled out because the Nd virion was internalized in cells as efficiently as the MVMp, since it could not be removed by NAs upon 1 h adsorption (Supplementary Figure S3B) and also because the IF confocal staining consistently localized the adsorpted Nd and MVMp virions inside the cells (Figures 6, 7C, Supplementary Figures S4, S5). Therefore, the restrictions on the infection by capsid contacts with inhibitory sia pseudoreceptors are exerted intracellularly.

In the restrictive infections, as those performed with Nd, most internalized virions were visualized, scattered across the cytosol with no apparent traffic fate (Figure 6, Supplementary Figure S4, *upper panels*). In contrast, in those permissive infections leading to high gene expression, as MVMp in NB324K and Nd in U373MG upon treatment with low doses of α2-3 S or α2-3,6,8 NAs, the virions showed a striking pattern of accumulation in clusters that could be localized by confocal IF within the endosomal contour delineated by the EEA-1 early marker (Figure 6, Supplementary Figure S4, *lower panels*). This Nd phenotype resulting from the NA treatments implies that a lower density in the sia glycan network on the cell surface facilitates the virus traffic to, and its accumulation in, the endosome. The characterization of the endosomal compartment where the incoming MVM virions cluster along the infectious entry route deserves further research. Furthermore, deciphering the precise composition of the glycans acting as functional sia receptors or inhibitory sia pseudoreceptors may provide a mechanistic explanation for the phenomenon of virion clustering that triggers the infection. It is worth mentioning that our findings relate to the capacity of gangliosides, with terminally attached sia, to classify extracellular cargos for uptake and trafficking early in the endocytic pathway (Ravindran et al., 2013). Based on our experimental observations, it could be hypothesized that the neuraminidase activity carried as a structural component by influenza (McAuley et al., 2019) and many other viruses entering by endocytosis may be required to cleave interacting gangliosides and sia glycans to facilitate virus traffic to the endosome.

**Figure 8:**
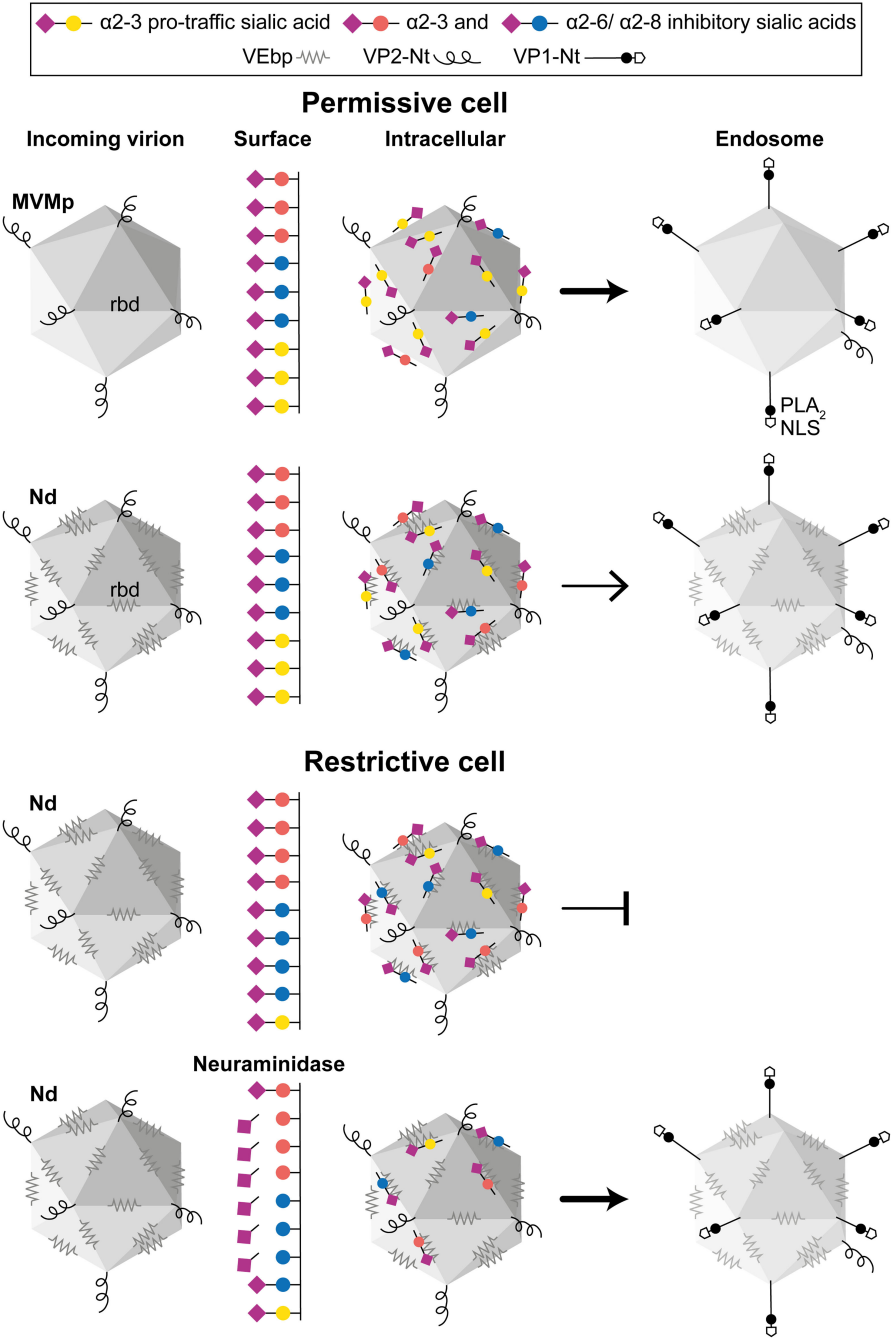
The role of capsid–sia interaction on parvovirus tropism. The figure illustrates a general model on parvovirus entry on the basis of the MVMp and chimeric MVM-Nd infection of human permissive (fibroblasts) and restrictive (GBM)-transformed cells, harboring different surface composition of pro-traffic α2-3 and inhibitory α2-3 and α2-6/α2-8-linked sia. **(Upper)** Both virions similarly bind to sia on the permissive cell surface, but the repertoire of sia moieties recognized by the specific residues of the rbd impacts the traffic to the endosome of the internalized virion. **(Lower)** Nonpermissive cells with higher proportion of inhibitory sia inhibit infection by precluding viral traffic to the endosome, which can be relieved by NA removal of inhibitory sia(s). For simplicity, only the Nd interaction has been illustrated here. In the productive infectious entry, viral capsids reach the low endosomal pH undergoing a drastic structural rearrangement that involves a *de novo* exposure and cleavage of the VP2-Nter of some protein subunits, which triggers the exposure of the VP1-Nter sequence. The VP1-Nter sequence harbors endosomal breaching membrane and nuclear localization sequences. rbd, Receptor binding domain in a dimple at the 2x axis; PLA_2_, phospholipase; NLS, nuclear localization sequence.

Several reports indicated that, despite the small size and simple T1 icosahedral structure, the MVM virion is a functionally complex macromolecular entity which undertakes essential capsid configuration rearrangements along the entry process to infect, including a *de novo* externalization of some VP2-Nt sequences through the 5x channel, their quantitative endosomal cleavage, and the subsequent externalization of the long VP1-specific Nt sequence (Farr et al., 2006; Mani et al., 2006; Sánchez-Martínez et al., 2012; Castellanos et al., 2013; reviewed in Ros et al., 2017). In tight consistency, the increased infection of the Nd virion by the NAs treatments (Figure 5) that significantly enhanced the endosomal accumulation of the incoming virion (Figure 6 and Supplementary Figure S5C) quantitatively correlated with the extent of VP2-Nt cleavage (Figure 7B) and the VP1-Nt exposure in the endosomal clusters (Figure 7C). Thus, the type of occupancy of the 2x receptor binding domain (rbd) of MVM capsid by sia moieties, which may act as functional receptors or nonproductive attachment factors or pseudoreceptors, impacts the capsid structural rearrangement required for the infectious entry (modeled in Figure 8).

In summary, our rational approach to replacing peptides in functional domains of the MVM icosahedral capsids yielded novel insights into the complex parvovirus–receptor interaction. The finding that an attachment to inhibitory glycans impacts the intracellular viral access to the endosome, where a drastic structural capsid transition onset infection opens the possibility to manipulate parvovirus tropism through intervening the cell type-dependent sia networks. This newly recognized determinant of MVM tropism may also enlighten the understanding of other viral systems in which the modulation of intracellular traffic by sia moieties may regulate cell entry, tropism, and host range.

## Data availability statement

The original contributions presented in the study are included in the article/Supplementary material, further inquiries can be directed to the corresponding author/s.

## Author contributions

TC-L, EG, and CS-M performed the experiments. TC-L constructed the figures. TC-L and JA analyzed the data. JA designed the research and wrote the manuscript. All authors contributed to the article and approved the submitted version.

## Supplementary material

Supplementary Figure S1 Tropism of the MVMp and Nd virions for NB324K and U373MG cells. The figure shows representative examples of IF staining showing a relative higher proportion of NS1+ in U373MG *vs*. NB324K cells upon inoculations with the Nd and NdPs virions. Numbers in parenthesis show the amount of virion inoculated as HAU. Scale bar, 50 μm.

Supplementary Figure S2 Cytometric analysis of NS1 expression in NB324K cells treated with α-2-3,6,8-sia cleaving neuraminidase. **(Upper)** relative NS1 expression values at the indicated doses of NA in MVMp- and Nd-infected cells sampled at 20 hpi; **(Lower)** example of the cytometric results obtained for both viruses with the 0.18 units NA doses. Infections and NA treatments were performed as in Figure 5.

Supplementary Figure S3 Affinity of attachment and internalization of the MVMp and Nd virions in human-transformed cells. **(A)** Binding affinity to the cell surface. MVMp and Nd adsorption kinetics to human NB324K fibroblast and U373MG glioblastoma cells analyzed by WB at the indicated time points. The binding at 4°C of 8x10^12^ purified MVMp or Nd viral particles in 100 μl of PBS^++^ inoculated onto 10^5^ cells in a M24 well is shown. *Left:* bound structural proteins (VP1, VP2, and VP3). Right: quantitative binding values obtained from the WB corresponding to the means with standard error from three independent experiments. M, Mock; I, input virus. **(B)** Analysis of virion uptake. Purified MVMp and Nd virions attached to both cell types at 4°C were treated (+) or not treated (-) with the indicated neuraminidases at 37°C for 1 h (*two left lanes*), or infection allowed to proceed for 1 h at 37°C prior NA treatments (*two right lanes*). Samples from both types of experiments were subjected to WB developing with the α-VPs antibody. Representative results of the amount of VP proteins bound to cells quantitatively determined by densitometry are shown.

Supplementary Figure S4 Endocytic accumulation of virions at 4 hpi may be increased by neuraminidase. Confocal IF staining of MVM capsid (α-MVM capsid polyclonal antibody) and the early endosomal (mouse anti-EEA1 antibody) of cells inoculated with 8x10^12^ purified MVMp or Nd virions per 10^5^ cells at 37 °C. Neuraminidase treatments were performed with 2.5x10^−2^ U/μl of α-2-3-NA and 10 x10^−2^ U/μl of α-2-3,6,8-NA. Samples were fixed at 4 hpi, and endosomes showing clusters of MVM capsid were quantitated as explained in Materials and Methods. Values correspond to the mean with standard errors from at least three fields of cells (N = 10^2^). Statistics is comparing the untreated to the NA-treated cells for each type of virion. Significance: ^*^*p* < 0.05; ^**^*p* < 0.01; ^***^*p* < 0.001. Scale bar, 50 μm.

Supplementary Figure S5 High-resolution confocal analysis of virion endocytic accumulation. Representative analysis of the distribution of viral capsids by sum projections of confocal image slices (z-tacks) in orthogonal views. The figure illustrates the clustering of incoming viral capsids in the endosome at 4 hpi in the MVMp infection of NB324K cells **(A)** and provoked by the NA treatment in Nd-infected U2373MG cells **(C)**, or capsids scattered across the cytosol in the respective negative controls **(B, D)**. Scale bar, 10 μm.
