# Correction: Intracellular virion traffic to the endosome driven by cell type specific sialic acid receptors determines parvovirus tropism

**DOI:** 10.3389/fmicb.2025.1640264

**Published:** 2025-07-02

**Authors:** Tania Calvo-López, Esther Grueso, Cristina Sánchez-Martínez, José M. Almendral

**Affiliations:** ^1^Centro de Biología Molecular Severo Ochoa (CSIC-UAM), Madrid, Spain; ^2^Departamento de Biología Molecular, Universidad Autónoma de Madrid, Madrid, Spain

**Keywords:** icosahedral capsid engineering, parvovirus, VEGF, tropism, sialic acid, virus entry and traffic, endosome, capsid structural transition

In the published article, there was an error in the legend for Figure 5 as published. “8 x 10^12^ viral particles per 10^5^ cells” instead of “8 x 10^9^ viral particles per 10^5^ cells” was written.

The corrected legend appears below.

**Figure 5**. Sialic acids distinctly contribute to MVMp and Nd virions binding and infection of human-transformed cells. The figure illustrates the effect of the α-2-3-NA and the α-2-3,6,8-NA sia cleaving neuraminidases on the binding and infection of MVMp and chimeric Nd virions in human-transformed NB324K fibroblasts and U373MG glioblastoma cells. Cell monolayers were inoculated with equivalent amounts of purified virions (8 x 10^9^ viral particles per 10^5^ cells) in the presence of the indicated concentrations of (left) α-2-3-NA and (right) α-2-3,6,8-NA sia cleaving neuraminidases. Samples were quantitatively analyzed in WB (see Materials and Methods) for: **(A, B)** Binding, developing for the structural proteins (VP1, VP2, VP3) after 1 h adsorption; and **(B, C)** productive infection, developing for the viral NS1 protein expression at 20 hpi. Each experiment was seeded and performed entirely in parallel for the **(A, C)** and **(B, D)** determinations. Each point of the graphs with standard errors represents the average obtained from three to seven independent experiments. Protein density values were normalized in each experiment and means with standard deviations were obtained from the normalized values. a.u. Relative arbitrary units of densitometry. Significance: ^*^p < 0.05, ^**^p < 0.01, ^***^p < 0.001. As above, the arrowhead marks a VP-related protein present in all cultured cell samples.

In the published article, there was an error in the legend for **Figure 6** as published. “8 x 10^12^ viral particles per 10^5^ cells” instead of “8 x 10^9^ viral particles per 10^5^ cells” was written.

The corrected legend appears below.

**Figure 6**. Capsid contacts with sia(s) modulate intracellular parvovirus traffic to the endosome. Effect of capsid–sia contacts in viral traffic to the endosome. The figure illustrates confocal IF staining of MVM capsid (α-MVM capsid polyclonal antibody) and the early endosomal (mouse anti-EEA1 antibody) of cells inoculated with equivalent amounts of purified virions (8 × 10^9^ viral particles per 10^5^ cells) adsorpted at 4°C (0 hpi) and further incubated 1 h at 37°C (1 hpi). Treatments were performed with 2.5 × 10^−2^ U/μl of α-2-3-NA and 10 × 10^−2^ U/μl of α-2-3,6,8-NA sia cleaving neuraminidases. The number of endosomes showing accumulation as clusters of MVM capsid was quantitated as explained in Materials and Methods. Values correspond to the mean with standard errors obtained from three fields (*N* = 10^2^ cells). Statistics was obtained comparing the untreated to the NA-treated cell monolayers. Significance: ^**^p < 0.01; ^***^p < 0.001. Scale bar, 50 μm.

A correction has been made to **Materials and methods**, *Virus titration*. Paragraph 2, sentence 2.

This sentence previously stated:

“ This method yielded 1.52 × 10^14^ viral particles per μg taking 3,970 kDa as the MW of the T1 MVM virion, based on the size of the VP1 and VP2 protein subunits (Gardiner and Tattersall, 1988), and their assembly stoichiometry (Riolobos et al., 2006).”

The corrected sentence appears below:

This method yielded 1.52 × 10^14^ viral particles per mg taking 3,970 kDa as the MW of the T1 MVM virion, based on the size of the VP1 and VP2 protein subunits (Gardiner and Tattersall, 1988), and their assembly stoichiometry (Riolobos et al., 2006).

The original article has been updated.

