# Supplementary figures and images for: Intracellular virion traffic to the endosome driven by cell type specific sialic acid receptors determines parvovirus tropism
Source: Front Microbiol. 2023 Jan 23;13:1063706. doi: 10.3389/fmicb.2022.1063706 (PMC9899843; doi:10.3389/fmicb.2022.1063706)

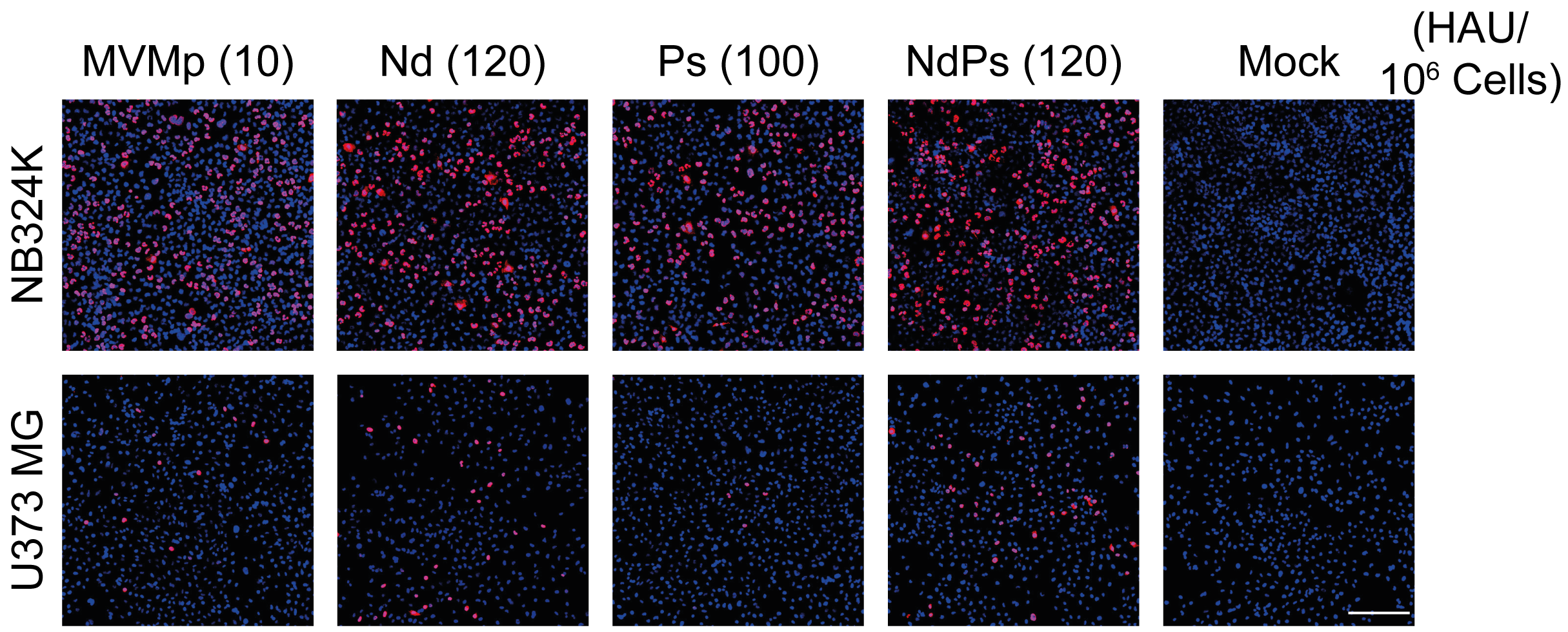

Supplement: Supplementary Figure S1 — Tropism of the MVMp and Nd virions for NB324K and U373MG cells. The figure shows representative examples of IF staining showing a relative higher proportion of NS1+ in U373MG vs. NB324K cells upon inoculations with the Nd and NdPs virions. Numbers in parenthesis show the amount of virion inoculated as HAU. Scale bar, 50 μm. [file Image_1.TIF]

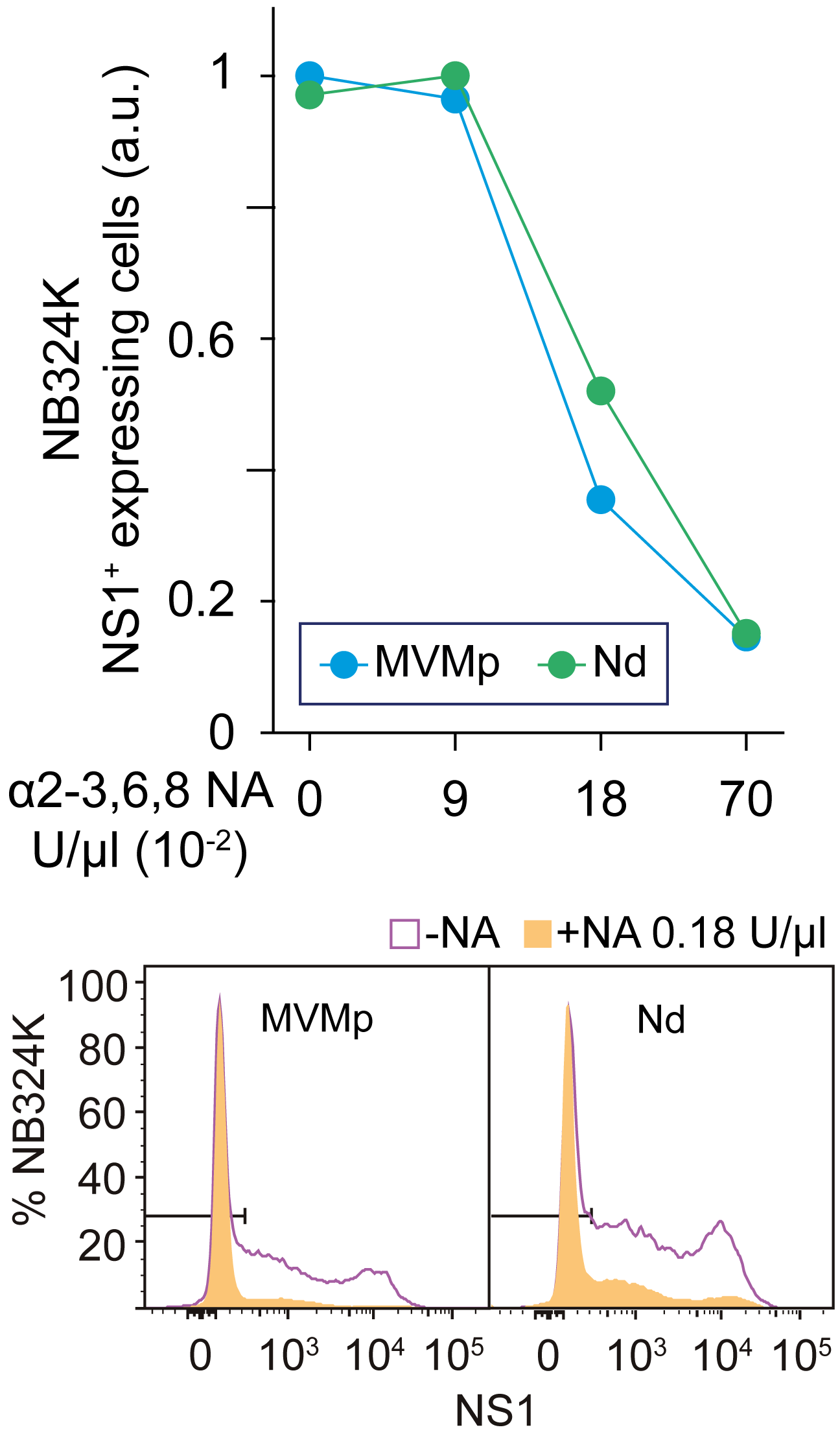

Supplement: Supplementary Figure S2 — Cytometric analysis of NS1 expression in NB324K cells treated with α-2-3,6,8-sia cleaving neuraminidase. (Upper) relative NS1 expression values at the indicated doses of NA in MVMp- and Nd-infected cells sampled at 20 hpi; (Lower) example of the cytometric results obtained for both viruses with the 0.18 units NA doses. Infections and NA treatments were performed as in Figure 5. [file Image_2.TIF]

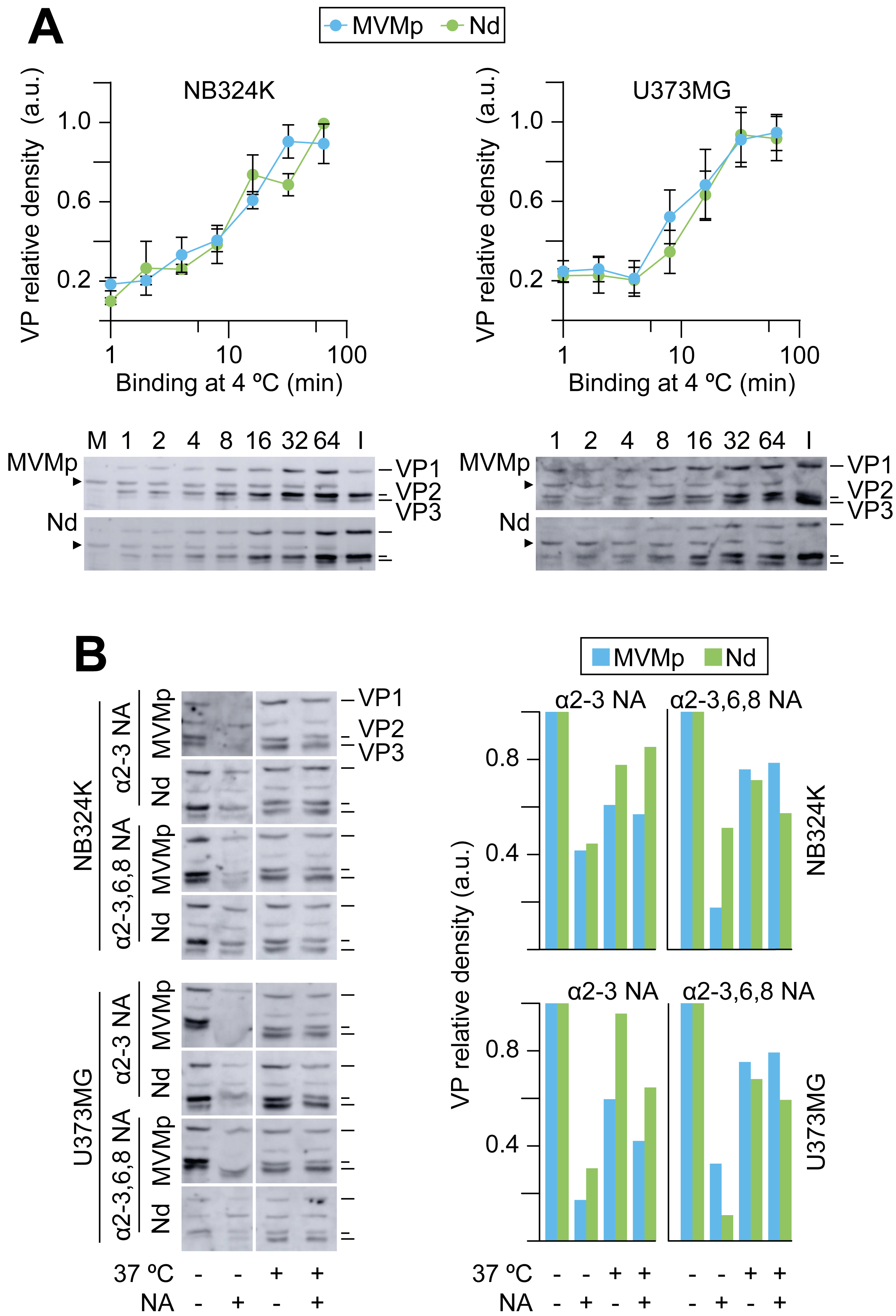

Supplement: Supplementary Figure S3 — Affinity of attachment and internalization of the MVMp and Nd virions in human-transformed cells. (A) Binding affinity to the cell surface. MVMp and Nd adsorption kinetics to human NB324K fibroblast and U373MG glioblastoma cells analyzed by WB at the indicated time points. The binding at 4°C of 8x10^12^ purified MVMp or Nd viral particles in 100 μl of PBS++ inoculated onto 105 cells in a M24 well is shown. Left: bound structural proteins (VP1, VP2, and VP3). Right: quantitative binding values obtained from the WB corresponding to the means with standard error from three independent experiments. M, Mock; I, input virus. (B) Analysis of virion uptake. Purified MVMp and Nd virions attached to both cell types at 4°C were treated (+) or not treated (-) with the indicated neuraminidases at 37°C for 1 h (two left lanes), or infection allowed to proceed for 1 h at 37°C prior NA treatments (two right lanes). Samples from both types of experiments were subjected to WB developing with the α-VPs antibody. Representative results of the amount of VP proteins bound to cells quantitatively determined by densitometry are shown. [file Image_3.TIF]

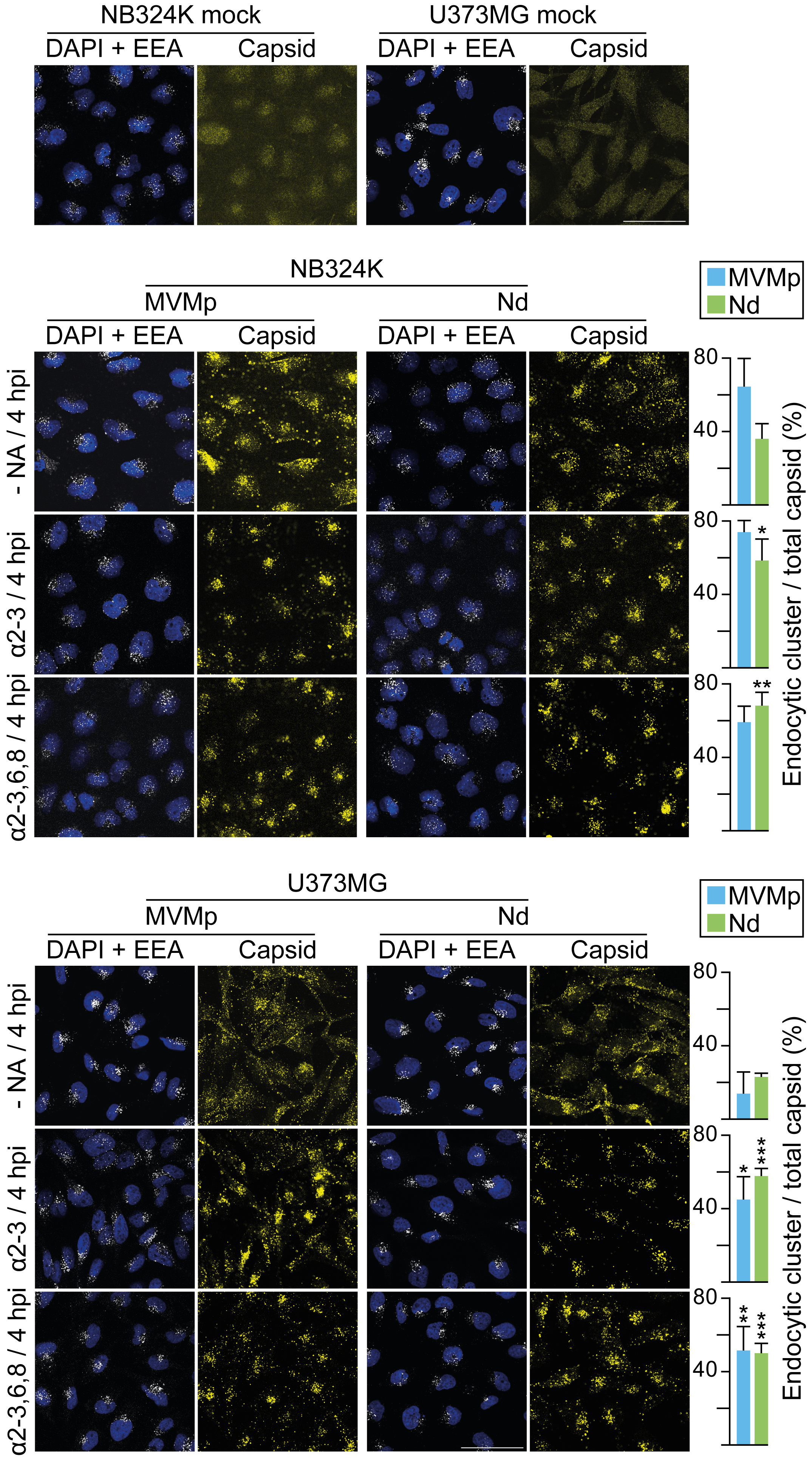

Supplement: Supplementary Figure S4 — Endocytic accumulation of virions at 4 hpi may be increased by neuraminidase. Confocal IF staining of MVM capsid (α-MVM capsid polyclonal antibody) and the early endosomal (mouse anti-EEA1 antibody) of cells inoculated with 8x10^12^ purified MVMp or Nd virions per 105 cells at 37 °C. Neuraminidase treatments were performed with 2.5x10^−2^ U/μl of α-2-3-NA and 10 x10−2 U/μl of α-2-3,6,8-NA. Samples were fixed at 4 hpi, and endosomes showing clusters of MVM capsid were quantitated as explained in Materials and Methods. Values correspond to the mean with standard errors from at least three fields of cells (N = 102). Statistics is comparing the untreated to the NA-treated cells for each type of virion. Significance: *p < 0.05; **p < 0.01; ***p < 0.001. Scale bar, 50 μm. [file Image_4.TIF]

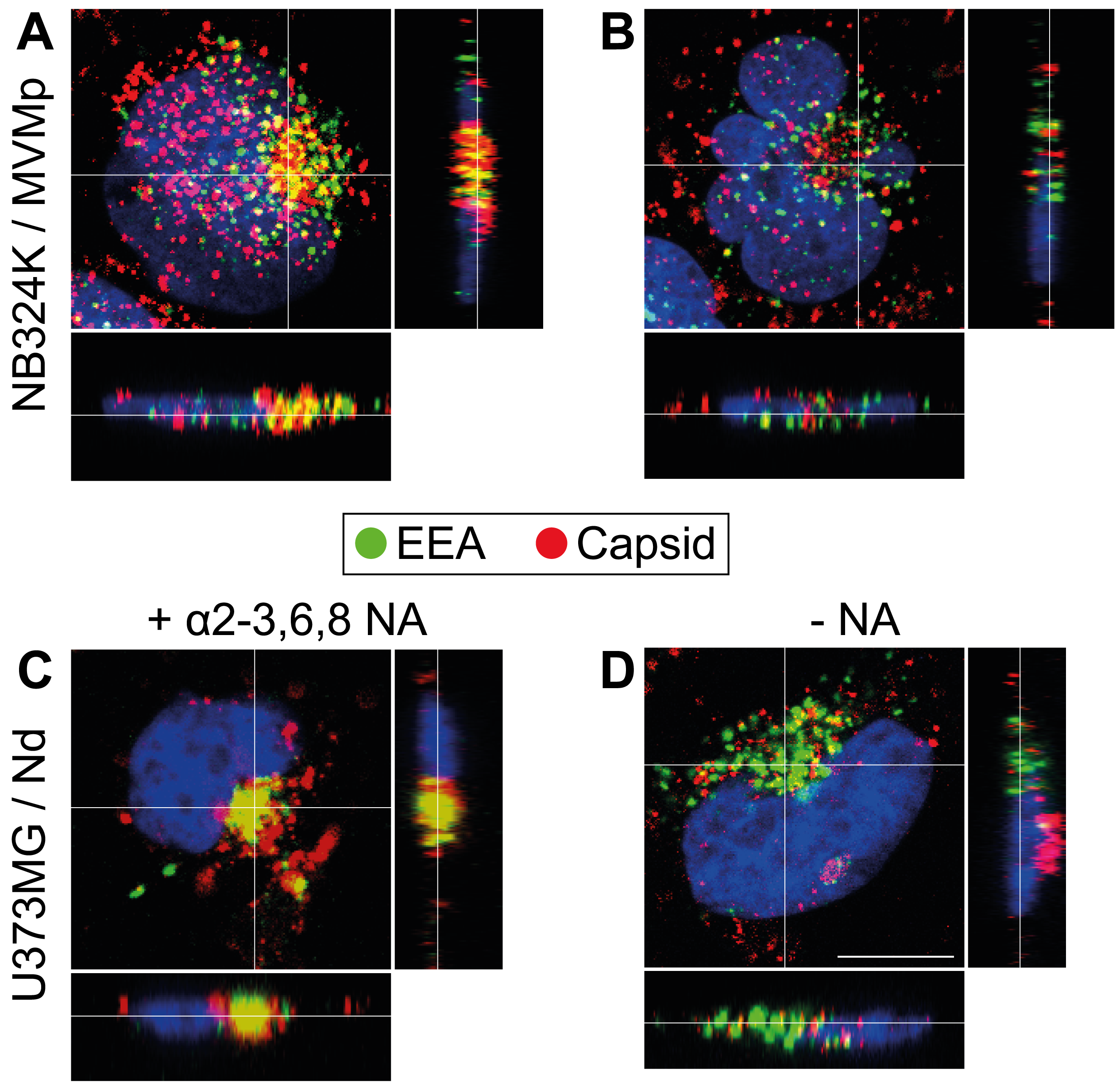

Supplement: Supplementary Figure S5 — High-resolution confocal analysis of virion endocytic accumulation. Representative analysis of the distribution of viral capsids by sum projections of confocal image slices (z-tacks) in orthogonal views. The figure illustrates the clustering of incoming viral capsids in the endosome at 4 hpi in the MVMp infection of NB324K cells (A) and provoked by the NA treatment in Nd-infected U2373MG cells (C), or capsids scattered across the cytosol in the respective negative controls (B, D). Scale bar, 10 μm. [file Image_5.TIF]
